# Targeting novel LSD1-dependent ACE2 demethylation domains inhibits SARS-CoV-2 replication

**DOI:** 10.1038/s41421-021-00279-w

**Published:** 2021-05-24

**Authors:** Wen Juan Tu, Robert D. McCuaig, Michelle Melino, Daniel J. Rawle, Thuy T. Le, Kexin Yan, Andreas Suhrbier, Rebecca L. Johnston, Lambros T. Koufariotis, Nicola Waddell, Emily M. Cross, Sofiya Tsimbalyuk, Amanda Bain, Elizabeth Ahern, Natasha Collinson, Simon Phipps, Jade K. Forwood, Nabila Seddiki, Sudha Rao

**Affiliations:** 1 Gene Regulation and Translational Medicine Laboratory, QIMR Berghofer Medical Research Institute, Brisbane, QLD Australia; 2 The Inflammation Biology Group, QIMR Berghofer Medical Research Institute, Brisbane, QLD Australia; 3 Medical Genomics, QIMR Berghofer Medical Research Institute, Brisbane, QLD Australia; 4 School of Biomedical Sciences, Charles Sturt University, Wagga Wagga, NSW Australia; 5 Department of Medical Oncology, Monash Health, Clayton, VIC Australia; 6 School of Clinical Sciences, Monash University, Clayton, VIC Australia; 7 Molecular Parasitology Laboratory, QIMR Berghofer Medical Research Institute, Brisbane, QLD Australia; 8 Respiratory Immunology Laboratory, QIMR Berghofer Medical Research Institute, Brisbane, QLD Australia; 9 U955, Equipe 16, Créteil, France; 10 Université Paris-Est Créteil, Faculté de médecine, Créteil, France; 11 Vaccine Research Institute (VRI), Créteil, France

**Keywords:** Epigenetics, Immunology, Post-translational modifications

## Abstract

Treatment options for COVID-19 remain limited, especially during the early or asymptomatic phase. Here, we report a novel SARS-CoV-2 viral replication mechanism mediated by interactions between ACE2 and the epigenetic eraser enzyme LSD1, and its interplay with the nuclear shuttling importin pathway. Recent studies have shown a critical role for the importin pathway in SARS-CoV-2 infection, and many RNA viruses hijack this axis to re-direct host cell transcription. LSD1 colocalized with ACE2 at the cell surface to maintain demethylated SARS-CoV-2 spike receptor-binding domain lysine 31 to promote virus–ACE2 interactions. Two newly developed peptide inhibitors competitively inhibited virus–ACE2 interactions, and demethylase access to significantly inhibit viral replication. Similar to some other predominantly plasma membrane proteins, ACE2 had a novel nuclear function: its cytoplasmic domain harbors a nuclear shuttling domain, which when demethylated by LSD1 promoted importin-α-dependent nuclear ACE2 entry following infection to regulate active transcription. A novel, cell permeable ACE2 peptide inhibitor prevented ACE2 nuclear entry, significantly inhibiting viral replication in SARS-CoV-2-infected cell lines, outperforming other LSD1 inhibitors. These data raise the prospect of post-exposure prophylaxis for SARS-CoV-2, either through repurposed LSD1 inhibitors or new, nuclear-specific ACE2 inhibitors.

## Introduction

COVID-19 remains a persistent and aggressive pandemic. Even at the asymptomatic stage, individuals infected with SARS-CoV-2 can remain infectious and shed the virus over an extended duration^1–3^. Despite some progress in the management of severe disease, with remdesivir FDA approved for hospitalized patients, and corticosteroids showing efficacy in severely ill patients^4,5^, the disease still has a high morbidity and mortality burden. Effective mass vaccination will take time to deliver, and not everyone will respond to or take vaccines. Furthermore, attempts to re-purpose old drugs or trial new agents to treat COVID-19 have been disappointing: hydroxychloroquine showed a lack of effect or even increased harm^6^, and targeted anti-IL-6 monoclonal antibody (mAb) therapy with sarilumab and tocilizumab has shown mixed results in clinical studies^7,8^. There is still a clinical need for new drugs to treat COVID-19 from initial virus exposure through to severe fulminant disease.

SARS-CoV-2 depends on and uses ACE2, a type I transmembrane metallocarboxypeptidase, as a cellular entry receptor, together with the serine protease TMPRSS2^9,10^. ACE2 is expressed in the lung, kidney, and gastrointestinal tract^11,12^. It has therefore become a critical therapeutic target in COVID-19, and a soluble form of ACE2, which binds to the spike (S) domain of SARS-CoV-2, prevents the virus from binding to cell membrane-bound ACE2 and infecting the cell, at least in vitro^13–15^. Furthermore, ACE2-targeting mAbs blocked entry of vesicular stomatitis virus pseudotypes expressing the SARS-CoV-2 spike protein, and camostat mesylate, which targets TMPRSS2, also partially blocked SARS-CoV-2-S-driven entry into Caco-2 cells^9^. While ACE2 appears to be a promising therapeutic target, it also has immunoprotective functions in lung tissues, guarding against inflammation-induced damage and severe acute lung failure in acute respiratory distress syndrome (ARDS)^16,17^. Therefore, any therapeutic targeting of ACE2 must balance its capacity to facilitate viral entry with its desirable immunoprotective effects.

SARS-CoV-2 is a single-stranded, positive-sense RNA virus. Recent data highlight that SARS-CoV-2, like many other RNA viruses, utilizes the importin nuclear shuttling machinery during infection to hijack host cell transcription and responses^18,19^. Several studies suggest that targeting the importin-mediated nuclear transport machinery may be critical for inhibiting SARS-CoV-2 infection. First, studies on SARS-CoV proteins have revealed a role for importin-α during infection in nucleocytoplasmic shuttling of the SARS-CoV nucleocapsid protein and subsequent host cell division^20–24^. Furthermore, the SARS-CoV accessory protein ORF6 has been shown to inhibit the antiviral activity of STAT1^25^. Finally, recent studies show that ivermectin, which inhibits the importin pathway, inhibits SARS-CoV-2 replication^26^.

Lysine-specific demethylase 1 (LSD1/KDM1A) is an H3K4/H3K9 de-methylase that also targets non-histone proteins including p53, DMNT1, and STAT3^27–29^. In its chromatin-modifying role, LSD1 selectively catalyzes the removal of mono-methylated and di-methylated groups from H3K4 and H3K9, with H3K4 methylation generally associated with gene activation and H3K9 methylation with repression^30,31^. LSD1 also plays a critical role in viral infections, and its expression is required for successful replication of both RNA (HIV) and DNA (HSV) viruses^32,33^. LSD1 inhibition induces the IFNβ/RIG/MDA5 viral mimicry pathway, inducing immunogenicity, suggesting that LSD1 re-programming could be important in mounting successful host anti-viral responses^34^. We therefore decided to investigate the role of LSD1 in SARS-CoV-2 infection.

We show for the first time that LSD1 is induced by and tightly couples to ACE2 following SARS-CoV-2 infection at both the cell membrane and in intracellular compartments in SARS-CoV-2-susceptible cells and human primary airway epithelium in vitro. LSD1 is critical for the regulation of the ACE2–SARS-CoV-2 S cell surface interaction required for viral entry. LSD1 directly interacts with the cytoplasmic tail of ACE2, which harbors a novel nuclear localization sequence (NLS)/methylation domain. LSD1 demethylation favors interaction between ACE2 and importin-α, a key nuclear shuttling protein, increasing RNA Pol-II-coupled ACE2 in the nucleus of SARS-CoV-2-infected cells. Elucidating the NLS within ACE2 and the interplay between cytoplasmic ACE2 and importin-α allowed the development of a novel, durable, and cell permeable nuclear ACE2 inhibitor (NACE2i), which significantly inhibited SARS-CoV-2 cellular replication. Other LSD1 inhibitors also inhibited SARS-CoV-2 cellular replication, albeit to a lesser extent than NACE2i. Targeting the nuclear ACE2 axis does not abolish cell surface ACE2, thereby providing a novel way to retain immunoprotection while decreasing replication for post-exposure prophylaxis (PEP). Furthermore, repurposing existing LSD1 inhibitors may offer a rapid route to PEP during the pandemic.

## Results

### LSD1 is enriched at the cell surface and in intracellular compartments of ACE2-expressing SARS-CoV-2-infected cells

**Fig. 1:**
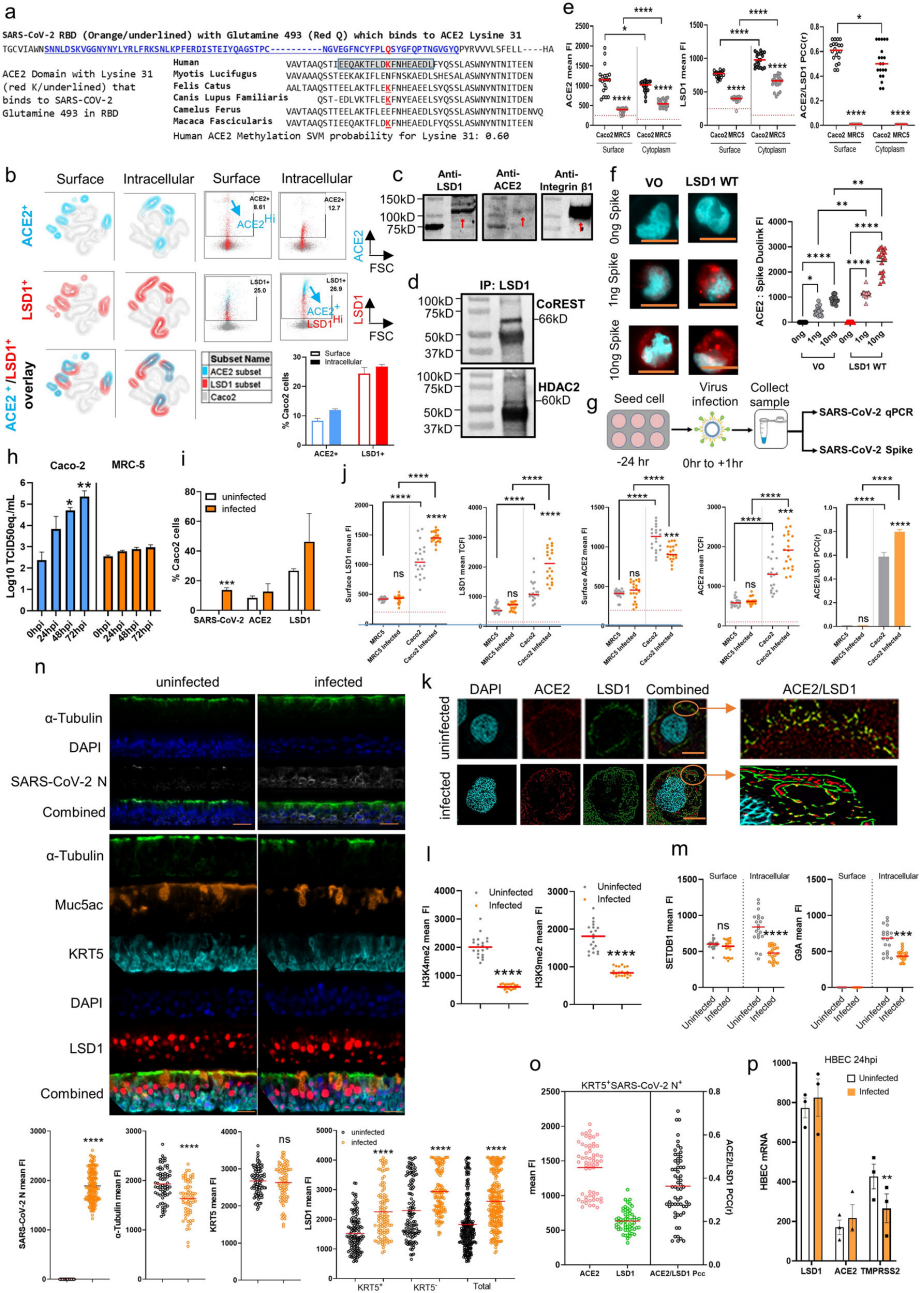
LSD1 is enriched at the cell surface and in intracellular compartments of ACE2-expressing cells. **a** The receptor-binding domain (RBD) sequence of SARS-CoV-2 showing the critical residue (Q; glutamine 493) that binds to ACE2 lysine 31 and the conservation of this sequence in different species. In silico prediction^35^ gave a probability of 0.7 of a methylation/demethylation signature at lysine 31. **b** FACS tSNE analysis of cell surface and intracellular expression of ACE2 and LSD1 in Caco-2 cells. The bar chart indicates the percentage ACE2^+^ or LSD1^+^ cells in the total Caco-2 population, also shown in the FACS dot plots (ACE2^+^ cells in blue and LSD1^+^ cells in red). Data are mean ± SEM (*n* = 3). **c** Western blot analysis of ACE2 and LSD1 cell surface expression by Caco-2 cells. Membrane lysates were analyzed by SDS-PAGE and blotted for ACE2, LSD1, and integrin-β1. **d** Western blot analysis of LSD1 IP samples. Following LSD1 IP of Caco-2 lysates, samples were analyzed by SDS-PAGE and blotted for CoREST (66kD) and HDAC2 (60kD). **e** Dot plot quantification of the fluorescence intensity (cell surface and cytoplasmic) of ACE2 and LSD1 in Caco-2 and MRC-5 cells. >50 cells were analyzed for each group. The Pearson correlation coefficient (PCC) was calculated for LSD1 and ACE2 colocalization (*n* = 20 cells analyzed). Mann-Whitney test: ^∗^*P* < 0.05, ^∗∗∗∗^*P* < 0.0001 denote significant differences. **f** Duolink^®^ proximity ligation assay measurements of protein interactions were performed on unpermeabilized Caco-2 cells transfected with either VO construct or an LSD1 WT plasmid followed by treatment with 0, 1, or 10 ng of SARS-CoV-2 spike protein. The Duolink assay produces a single bright dot per interaction within the cell. Representative images (top) are shown for ACE2 and SARS-CoV-2 Spike Duolink^®^. PLA signal intensity of the Duolink^®^ assay (bottom) is shown for average dot intensity (single Duolink dot). Data represents *n* = 20 cells, with significant differences calculated using the Kruskal-Wallis ANOVA (^∗^*P* < 0.05, ^∗∗^*P* < 0.01, ^∗∗∗∗^*P* < 0.0001). Representative images are shown with 10 µM scale bar in orange. **g** Schematic of SARS-CoV-2 infection assays. Caco-2 cells were seeded 24 h before the experiment. Then, cells were infected with SARS-CoV-2 (MOI 1.0). After 1 h viral adsorption incubation, the virus inoculum was removed and drug-free medium was added. Then, cell culture supernatants were harvested at 0, 24, 48, or 72 hpi (hour post-infection) and infected cells collected at 24, 48, or 72 hpi. Viral genomes were detected in the extracted RNA by qRT-PCR, and viral protein was quantified by digital pathology (ASI system). **h** qRT-PCR analysis to detect the growth kinetics of SARS-CoV-2 in Caco-2 and MRC-5 culture supernatants at the indicated time points after viral infection. The quantity of viral genomes is expressed as log10 TCID_50_ equivalents/mL. Data are mean ± SEM (*n* = 3). One-way ANOVA; ^∗^*P* < 0.05, ^∗∗^*P* < 0.01 denote significant differences. **i** FACS analysis of expression of SARS-CoV-2 N (nucleocapsid), ACE2, and LSD1 in uninfected vs. SARS-CoV-2-infected Caco-2 cells (*n* = 3, unpaired *t*-test: ^∗∗∗^*P* < 0.001). **j** Dot plot quantification of the fluorescence intensity (cell surface and cytoplasmic) of ACE2 and LSD1 in uninfected or SARS-CoV-2-infected CaCo-2 and MRC-5 cells. >50 cells were analyzed for each group. Mann–Whitney test: n.s. denotes non-significant, ^∗∗∗^*P* < 0.001, ^∗∗∗∗^*P* < 0.0001. The PCC was calculated to assess colocalization in MRC-5 or Caco-2 cells with/without infection (*n* = 20 cells analyzed). Data are mean ± SEM. Mann-Whitney test: n.s. denotes non-significant, ^∗∗∗∗^*P* < 0.0001 denote significant differences. **k** Representative image of uninfected or SARS-CoV-2-infected Caco-2 cells using the Andor WD Revolution Inverted Spinning Disk microscopy system. Cells were not permeabilized (surface), with immunostaining for ACE2, LSD1, and SARS-CoV-2 N (nucleocapsid). DAPI (blue) was used to visualize nuclei. Scale bar, 12 μm (inset). **l** Dot plot quantification of the fluorescence intensity of H3K9me2 and H3K4me2 in uninfected or SARS-CoV-2-infected Caco-2 cells. >50 cells were analyzed for each group. Mann–Whitney test: ^∗∗∗∗^*P* < 0.0001 denote significant differences. **m** Dot plot quantification of the fluorescence intensity (cell surface and intracellular) of SETDB1, G9A, and ACE2 in uninfected or SARS-CoV-2-infected Caco-2 cells. >50 cells were analyzed for each group. Mann-Whitney-test: n.s. denotes non-significant, ^∗∗∗^*P* < 0.001, ^∗∗∗∗^*P* < 0.0001 denote significant differences. **n** Representative image of uninfected or SARS-CoV-2 infected human biliary epithelial cells (HBECs) using the ASI digital pathology system and immunostaining for α-tubulin, Muc5ac, KRT5, LSD1, ACE2, and SARS-CoV-2 N (nucleocapsid). DAPI (blue) was used to visualize nuclei. Scale bar, 12 μm (inset). Dot plot quantification of the mean fluorescent intensity of α-tubulin, KRT5, and SARS-CoV-2 N (nucleocapsid), and the mean intracellular (cytoplasmic and nuclear combined) fluorescence intensity of LSD1 in uninfected or SARS-CoV-2-infected cells. *n* = 3, >20 cells were analyzed for each biological repeat. Mann–Whitney test: ^∗∗∗∗^*P* < 0.0001 denote significant differences. **o** Dot plot quantification of the fluorescence intensity of ACE2 and LSD1 in KRT5^+^SARS-CoV-2 N^+^ cells. The PCC was calculated for LSD1 and ACE2 colocalization. *n* = 3, >20 cells were analyzed for each biological repeat. **p** qRT-PCR analysis of *LSD1, ACE2*, and *TMPRSS2* mRNA expression in uninfected versus SARS-CoV-2-infected HBECs at 48 hpi. Data are mean ± SEM. (*n* = 3, unpaired *t*-test: **P < 0.01).

Many coronavirus proteins are modified by post-translational modifications (PTMs), and epigenetic enzymes fine-tune the regulation of critical proteins in response to environmental cues via methylation and demethylation PTMs^27–29^. Here, using an in silico PTM prediction approach^35^, we surveyed the ACE2 sequence to identify lysine residues likely to be methylated or demethylated. Lysine 31 was predicted to be susceptible to, and therefore likely to be regulated by, methylation/demethylation events based on a high score cut-off as defined by the in silico prediction tool^35^ (Fig. 1a). This residue is highly conserved across species and is critical for interactions with the receptor-binding domain (RBD) of the SARS-CoV-2 spike protein at glutamine 493 (Fig. 1a)^36–38^.

We hypothesized that LSD1 might be a post-translational modifier of ACE2 lysine 31 because: a) LSD1 is known to demethylate both histone and non-histone proteins such as p53, which alters interaction partners^27–29^; and b) bioinformatics analysis of LSD1 using TMpred^39^ revealed several high-scoring transmembrane domains suggesting a novel cell surface role, while other methyltransferases such as G9a or SETDB1 had low-scoring or no-scoring regions indicating low or no transmembrane potential (Supplementary Fig. S1a). We therefore examined whether LSD1 regulates ACE2 in SARS-CoV-2-susceptible Caco-2 (human colorectal adenocarcinoma) and SARS-CoV-2 non-susceptible MRC-5 (human lung fibroblast) cells^9^. Consistent with Chu et al.^40^, Caco-2 cells expressed significantly higher levels of *ACE2* and entry protease *TMPSSR2* transcript than MRC-5 cells (Supplementary Fig. S1b); furthermore, *LSD1* mRNA expression was also higher in Caco-2 cells. Using cell surface and intracellular flow cytometric analysis of LSD1 and ACE2 expression coupled with t-distributed stochastic neighbor embedding (tSNE) analysis, all ACE2^+^ Caco-2 cells co-expressed LSD1 both at the cell surface and within intracellular compartments, suggesting a novel role for LSD1 at the cell surface (Fig. 1b). ACE2, predominantly regarded as a transmembrane enzyme, showed higher expression levels at the cell surface than in intracellular compartments, while LSD1 showed higher expression within intracellular compartments, with only the LSD1^hi^ intracellular population displaying ACE2 co-expression (Fig. 1b). LSD1 and ACE2 expression was similar in MRC-5 cells but with much higher ACE2 expression at the cell surface (22.9%) than intracellularly (5.4%) (Supplementary Fig. S1c), suggesting that ACE2 localization might be associated with the SARS-CoV-2-resistant phenotype. To further confirm the co-existence of LSD1 and ACE2 at the cell surface of Caco-2 cells, membrane proteins were extracted from Caco-2 cells and subjected to western blot analysis. The data confirmed ACE2 and LSD1 protein expression at the cell surface (Fig. 1c), with the presence of integrin B as a positive control confirming membrane protein extraction (Fig. 1c). LSD1 interactions with CoREST and HDAC2 in Caco-2 total protein extracts were also analyzed via co-immunoprecipitation with antibodies against LSD1 and, consistent with previous data, LSD1 associated with CoREST and HDAC2 (Fig. 1d).

We next visualized and quantified the co-existence of ACE2 and LSD1 in Caco-2 and MRC-5 cells by immunofluorescence imaging (Fig. 1e) and lamin B1 (nuclear/intracellular only) and integrin-β I (cell surface/cytoplasm only) to confirm the fidelity of our permeabilization protocols (Supplementary Fig. S1d). ACE2 and LSD1 mean fluorescent intensities (mFI) were higher at both the surface and within the cytoplasm of Caco-2 cells than MRC-5 cells, with cell surface LSD1 mFI substantially greater in Caco-2 than MRC-5 cells, consistent with flow cytometry results. Furthermore, ACE2 and LSD1 co-localized significantly more in Caco-2 cells in both compartments (Fig. 1e). LSD1 and ACE2 cell surface co-expression was also confirmed in the human non-small cell lung carcinoma line H1299 and the human breast cancer line MDA-MB-231 (Supplementary Fig. S1e).

To further investigate the relationship between LSD1 and ACE2 co-expression, MRC-5 and Caco-2 cells were transfected with either vector only (VO) or wildtype LSD1 (LSD1 WT) plasmids (Supplementary Fig. S1f). Transfection of Caco-2 and MRC-5 cells with LSD1 WT increased the LSD1 mFI compared to VO controls (Supplementary Fig. S1f) and, mirroring this, ACE2 mFI increased in both cell lines, with Caco-2 cells showing the greatest upregulation of ACE2 (Supplementary Fig. S1f). Taken together, LSD1 and ACE2 are enriched and co-exist at both the cell surface and intracellularly in SARS-CoV-2-susceptible cell lines. Consistent with the above findings, Caco-2 cells transfected with LSD1 WT and treated with SARS-CoV-2 spike protein showed significantly increased co-localization of ACE2, and the spike protein at the cell surface compared to VO-treated cells using a proximity ligation assay (Fig. 1f). Overall, this combination of approaches show that LSD1 co-exists with ACE2 at the cell surface and this association increases interactions with the SARS-CoV-2 spike protein.

We next examined changes in LSD1 and ACE2 following SARS-CoV-2 infection in Caco-2 and MRC-5 cells (Fig. 1g). In agreement with Chu et al.^40^, there was a significant increase in viral replication over 72 h in Caco-2 cells, while MRC-5 cells were resistant to SARS-CoV-2 infection with negligible virus replication at 72 hpi (Fig. 1h). At 48 hpi, 13.7% of Caco-2 cells were infected with SARS-CoV-2, with a trend towards increased infection of LSD1^+^ cells (Fig. 1i). Similarly, there was a trend towards increased *LSD1* gene expression at 48 hpi and a decrease in *ACE2* transcription, consistent with previous studies^12^ (Supplementary Fig. S1g).

To gain a better understanding of LSD1 and ACE2 co-localization in SARS-CoV-2 susceptible and resistant cells, immunofluorescence analysis using previously established controls (see “Materials and methods” section) was employed to assess the dynamics of expression of both proteins after viral infection. There was an increase in cell surface LSD1 mFI in Caco-2 cells after infection (Fig. 1j). Additionally, intracellular LSD1 and ACE2 were significantly induced following infection (Fig. 1j), indicating translocation of ACE2 from the cell surface to intracellular compartments, in agreement with previous findings^28^. There was no significant change in ACE2 or LSD1 surface or intracellular expression in MRC-5 cells (Fig. 1j).

Super-resolution imaging (Andor spinning disc confocal microscopy) allows unprecedented visualization of proteins in different cellular compartments at the single cell level to confirming protein localization and interactions. Super-resolution imaging of infected and uninfected Caco-2 cells further supported co-localization of LSD1 and ACE2 at the cell surface of uninfected cells (Fig. 1k), and increased co-localization in SARS-CoV-2 infected cells (Fig. 1k).

Consistent with increased LSD1 expression and activity, there was a global decrease in H3K4me2 and H3k9me2 methylation following infection (Fig. 1l). Unlike LSD1, the closely related epigenetic enzymes G9A and SETDB1 showed no cell surface expression and decreased intracellular expression following SARS-CoV-2 infection (Fig. 1m), consistent with our in silico predictions of transmembrane domains (Supplementary Fig. S1a).

The bronchial epithelium is an important barrier tissue that is pathologically altered in a variety of viral infections^41^. To verify the phenotypes observed in Caco-2 cells, we next used primary human bronchial epithelial cells obtained from healthy donors to assess airway epithelial changes following SARS-CoV-2 infection. The cells were differentiated at air–liquid interfaces (ALIs) to generate muco-ciliated tissue containing specialized bronchial epithelial cell subtypes, including ciliated cells (α-Tub^+^), goblet cells (Muc5ac^+^), and basal cells (KRT5^+^)^42,43^. There was a significant increase in infectious virus over 48 h in HBECs (Supplementary Fig. S1h). We performed immunofluorescence and digital pathology analysis on ALI cells to assess changes in cell phenotype and LSD1 expression following SARS-CoV-2 infection (Fig. 1n). High levels of SARS-CoV-2 nucleocapsid protein were detected in HBECs after infection accompanied by downregulation of α-tubulin and upregulation of intracellular (nuclear and cytoplasmic) LSD1 (Fig. 1n). Ciliated cells have also been reported as a major SARS-CoV-2 target^44^. There were higher levels of cytoplasmic and nuclear LSD1 in KRT5^−^ non-basal cells such as α-tubulin-positive ciliated cells, which was further significantly increased upon infection. ACE2 has previously been shown to be enriched in KRT5^+^ basal cells in the ALI cell model^45^. Hence, we examined co-expression of LSD1 and ACE2 in our ALI cell model in permeabilized, SARS-CoV-2-infected cells. KRT5^+^SARS-CoV-2 N^+^ cells expressed high levels of ACE2, with co-localization of ACE2 and LSD1 in infected basal cells (Fig. 1o). Notably, however, *LSD1* and *ACE2* expression was comparable in uninfected and SARS-CoV-2-infected HBEC cells 24 hpi, whilst *TMRPSS2* expression significantly decreased upon infection (Fig. 1p).

### LSD1 inhibitors and novel cell permeable peptides inhibit ACE2–spike interactions and cellular entry of SARS-CoV-2

**Fig. 2:**
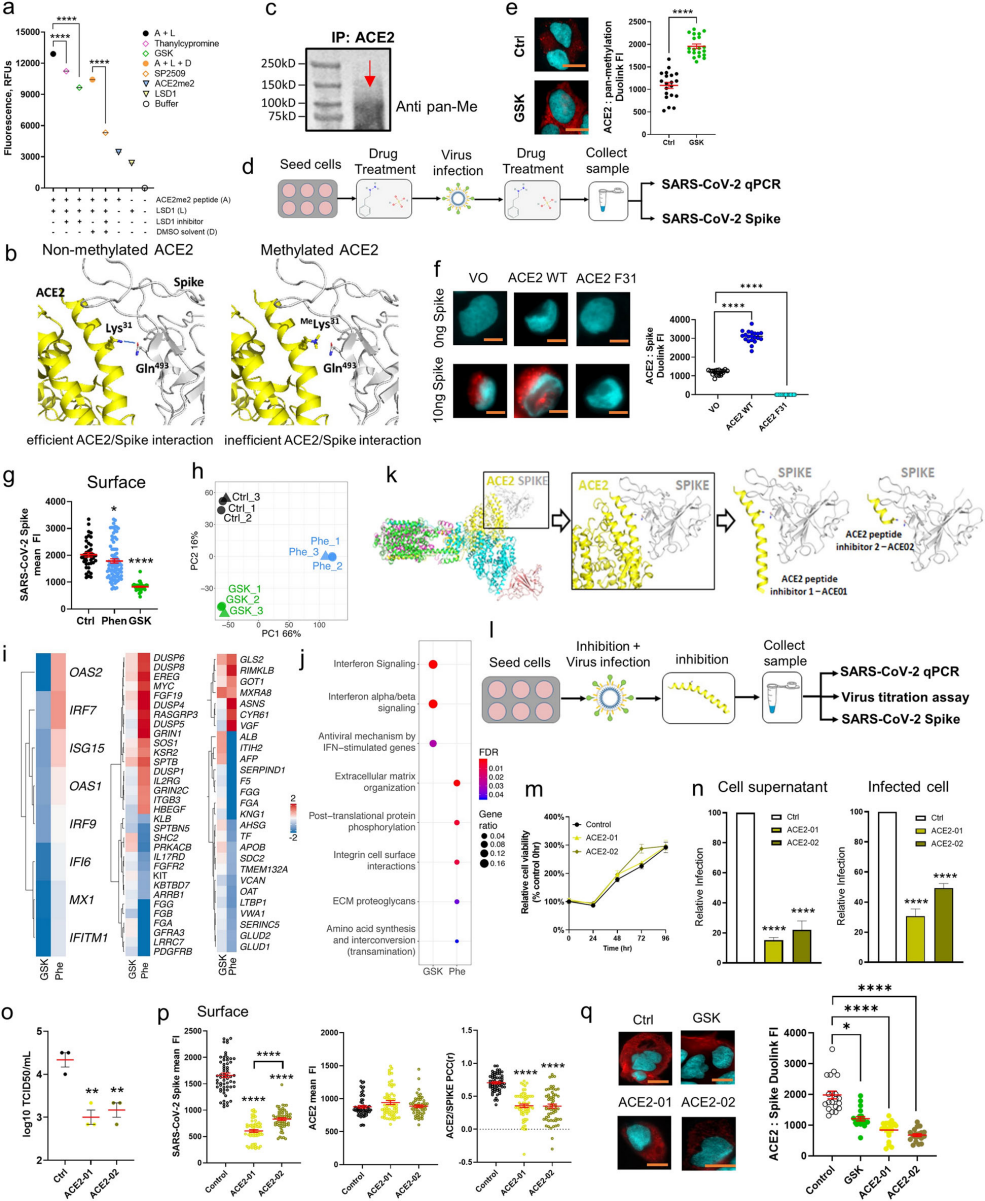
LSD1–ACE2 interactions and the effect of LSD1 on the spike protein. **a** Inhibition of ACE2me2 peptide demethylation by LSD1 inhibitors. LSD1 enzyme was incubated with tranylcypromine, GSK, SP2509, or DMSO for 30 min. The LSD1 activity in each sample was measured by a demethylation assay with ACE2me2 peptide. Fluorescence was measured at 40 s intervals on a Synergy H4 multi-mode plate reader. Statistical significance was calculated using one-way ANOVA, ^∗∗∗∗^*P* < 0.0001. **b** Structure of ACE2 bound to the SARS-CoV-2 spike domain (PDB 6M17). Binding of ACE2 and the spike domain involves a Lys^31^ (ACE2) and Gln^493^ (spike) interaction. ACE2 is shown in yellow in cartoon mode, and spike domain in gray. Residues are shown in stick format. Methylation of ACE2 Lys31 (right panel) would disrupt this interaction. **c** Western blot analysis of ACE2 IP samples. Following ACE2 IP of Caco-2 cell membrane extract lysates, samples were analyzed by SDS-PAGE and blotted for pan-methylation lysine. **d** Schematic of SARS-CoV-2 infection assays. Caco-2 cells were seeded 24 h before the experiment. Then, cells were treated with each drug component for 48 h followed by SARS-CoV-2 infection (MOI 1.0). After 1 h viral adsorption incubation, the virus inoculum was removed and drug-containing medium was added. Then, cell culture supernatants were harvested at 0, 24, or 48 hpi and infected cells were collected at 48 hpi. Detection of viral genomes in the extracted RNA was performed by qRT-PCR, and viral spike proteins were quantified by a digital pathology assay (ASI system). **e** Duolink^®^ proximity ligation assay measurements of protein interactions were performed on unpermeabilized Caco-2 cells infected with SASR-CoV-2 and treated with control or GSK. The Duolink assay produces a single bright dot per interaction within the cell. Representative images (left) are shown for ACE2 and pan-methylation lysine antibody. PLA signal intensity of the Duolink^®^ assay (right) is shown for average dot intensity (single Duolink dot) or overall cell intensity for each cell. Data represent *n* = 20 cells, with significant differences calculated with the unpaired *t*-test (^∗∗∗∗^*P* < 0.0001). Representative images are shown with 10 µM scale bar in orange. **f** Duolink^®^ proximity ligation assay measurements of protein interactions were performed on unpermeabilized Caco-2 cells transfected with VO, ACE2 WT, or ACE2 F31 plasmids and treated with 50 ng of SARS-CoV-2 spike protein. Samples were then probed with antibodies specific for ACE2 and SARS-CoV-2 spike protein. The Duolink assay produces a single bright dot per interaction within the cell. Representative images (left) are shown for ACE2 and SARS-CoV-2 Spike Duolink^®^. PLA signal intensity of the Duolink® assay (right) is shown for average dot intensity (single Duolink dot). Data represent *n* = 20 cells, with significant differences calculated with Kruskal–Wallis ANOVA (^∗∗∗∗^*P* < 0.0001). Representative images are shown with 10 µM scale bar in orange. **g** Dot plot quantification of the fluorescence intensity (cell surface) of SARS-CoV-2 spike protein in SARS-CoV-2-infected Caco-2 cells with phenelzine or GSK treatment. >50 cells were analyzed for each group and were quantified using the digital pathology assay (ASI system). Mann–Whitney test: ^∗^*P* < 0.05, ^∗∗∗∗^*P* < 0.0001 denote significant differences. **h** Principal component analysis (PCA) depicting transcriptional profiles for control, GSK, and phenelzine groups after batch effect removal. Experimental batches represented by different shapes (circles, batch 1; triangles, batch 2). **i** Heatmap of DEGs belonging to Reactome pathways: R-HSA-913531 interferon signaling (left); MAPK signaling-related pathways (R-HSA-5683057 MAPK family signaling cascades, R-HSA-5673001 RAF/MAP kinase cascade, and R-HSA-5684996 MAPK1/MAPK3 signaling; middle); and translation related pathways (R-HSA-70614 amino acid synthesis and interconversion (transamination), R-HSA-8957275 post-translational protein phosphorylation; right). The heatmap values depict the log_2_-fold change (logFC) of DEGs from treated cells compared with control cells (GSK vs. control and phenelzine vs. control). **j** Dot plot visualization of the top enriched Reactome pathways in treated cells compared to control cells. The dot color represents the false discovery rate (FDR) value for each enriched Reactome pathway and size represents the gene ratio. **k** Structure of ACE2 bound to the SARS-CoV-2 spike protein as depicted in **a**. Also depicted are the two peptide inhibitors targeting this region (ACE2-01, ACE2-02) and the interaction with the SARS-CoV-2 spike protein. **l** Schematic of SARS-CoV-2 infection. Caco-2 cells were seeded for 24 h and then infected with SARS-CoV-2 at MOI 1.0 in the presence of ACE2 peptide inhibitors (ACE2-01 or ACE2-02) for 1 h. The virus inoculum was removed and inhibitor-containing medium was added. Then, cell culture supernatants were collected at 0 or 48 hpi and infected cells were harvested at 48 hpi. Antiviral activity was assessed with three viral assays: SARS-CoV-2 qRT-PCR, median tissue culture infective dose assay (TCID_50_), and viral SPIKE protein quantified by digital pathology (ASI system). **m** Cell proliferation analysis of Caco-2 control and ACE2-01/ACE2-02-treated cells over a 96-h period. Proliferation was analyzed using WST-1 reagent and absorbance read after 2 h incubation. The graph depicts relative cell proliferation from three replicates expressed as a percentage of control cells (untreated, 0 h). Statistical significance was calculated using one-way ANOVA at each time point. **n** qRT-PCR analysis to detect replicates of SARS-CoV-2 RNA in Caco-2 culture supernatants and infected cells at the indicated time points post-infection. Relative infection was normalized to the uninfected control. Data represent mean ± SEM, *n* = 3. One-way ANOVA, ^∗∗∗∗^*P* < 0.0001 denotes significant differences. **o** TCID_50_ assay to measure infectious viral titers in the culture supernatants of infected cells. Data represent mean ± SEM, *n* = 3. One-way ANOVA, ^∗∗^*P* < 0.01 denotes significant differences. **p** Dot plot quantification of the fluorescence intensity (cell surface) of SARS-CoV-2 spike and ACE2 in SARS-CoV-2-infected Caco-2 cells with ACE2-01 or ACE2-02 treatment. >50 cells were analyzed in each group and were quantified by digital pathology (ASI system). The PCC was calculated for colocalization (*n* = 20 cells were analyzed). Mann–Whitney test: ^∗∗∗∗^*P* < 0.0001 denote significant differences. **q** Duolink^®^ proximity ligation assay measurements of protein interactions were performed on unpermeabilized Caco-2 cells infected with SARS-CoV-2 and treated with control, GSK, or ACE2-01 or ACE2-01 peptide inhibitors. The Duolink assay produces a single bright dot per interaction within the cell. Representative images (left) are shown for ACE2 and SARS-CoV-2 Spike Duolink^®^. PLA signal intensity of the Duolink® assay (right) is shown for average dot intensity (single Duolink dot). Data represent *n* = 20 cells, with significant differences calculated with Kruskal–Wallis ANOVA (^∗^*P* < 0.05, ^∗∗∗∗^*P* < 0.0001). Representative images are shown with 10 µM scale bar in orange.

The above data show that LSD1 associates with ACE2 at the cell surface following SARS-CoV-2 infection. Furthermore, ACE2 lysine 31 is predicted to undergo de-methylation/methylation (Fig. 1a) and interact with glutamine 493 in the SARS-CoV-2 spike protein receptor-binding domain (RBD)^37^. We therefore addressed the ability of LSD1 to directly de-methylate ACE2 at lysine 31 with LSD1 activity assays using peptides mimicking the methylated lysine 31 motif. To assess whether LSD1 inhibition reduced ACE2 demethylation at lysine 31, recombinant LSD1 protein alone or pre-incubated with different LSD1 inhibitors and di-methylated ACE2 peptide (Supplementary Table S1 and S2a) was used as a substrate to measure the demethylation reaction in an in vitro LSD1 activity assay. The peptide contained the motif predicted to undergo methylation/de-methylation using the in silico prediction software PSSme^34^ and also representing the binding region between glutamine 493 in the receptor-binding domain (RBD) of the SARS-CoV-2 spike protein and ACE2^37^: QAKTFLD{Lys(Me2)}FNHEAED (Supplementary Table S1), with a di-methylated lysine at position 31. LSD1 efficiently de-methylated the ACE2 peptide at lysine 31. Furthermore, tranylcypromine, a monoamine oxidase inhibitor (MAO) of LSD1^46^, significantly decreased ACE2 lysine 31 demethylation, and further decreases were observed in samples treated with GSK2879552 (GSK), an irreversible inhibitor of LSD1 catalytic activity (Fig. 2a). Interestingly, SP2509, an enzymatic^47^ and allosteric LSD1 inhibitor^48^, showed the highest inhibitory potency of all the LSD1 inhibitors in vitro (Fig. 2a).

As noted above, glutamine 493 in the SARS-CoV-2 spike protein RBD binds to lysine 31 of ACE2^37^. We therefore performed structural analysis and modeling to assess whether LSD1-mediated demethylation affects interactions between the spike RBD and ACE2 receptor, with methylation of ACE2 lysine 31 predicted to reduce, and de-methylation increase, the efficiency of glutamine 493 RBD binding (Fig. 2b). Membrane proteins were extracted from Caco-2 cells and subjected to co-immunoprecipitation with antibodies targeting ACE2 followed by western blotting with pan methyl lysine antibody. Methylated ACE2 was present at the cell surface (Fig. 2c and Supplementary Fig. S2b). Phenelzine and GSK have different modes of action: phenelzine can disrupt the nuclear LSD1/CoREST complex, while GSK is a significantly more potent inhibitor of LSD1 catalytic activity than phenelzine^49^. Caco-2 cells were pre-treated with previously optimized equivalent doses of inhibitors^49,50^; neither altered cell proliferation (Supplementary Fig. S2c), confirming that LSD1 inhibition does not affect cellular replication; furthermore, cell viability was consistent (>90%) between treatment groups up to 72 h (Supplementary Fig. S2d).

To determine whether GSK inhibited ACE2 demethylation at the cell surface of Caco-2 cells, cells were treated with inhibitor for 48 h followed by SARS-CoV-2 infection (MOI 1.0) for 1 h and culture in inhibitor-containing medium for up to 48 h (Fig. 2d). Using a proximity ligation assay to assess co-localization, GSK treatment indeed increased the association between ACE2 and pan-methylation lysine antibody in infected cells, suggesting that LSD1 modulates the methylation status of ACE2 (Fig. 2e).

To specifically examine the impact of lysine 31 demethylation on spike protein interactions, we next used plasmid constructs expressing either wild-type ACE2 or mutant ACE2 with a hypermethylation mimic (ACE2 K31F) replacing lysine 31 (with phenylalanine) to transfect Caco-2 cells subsequently treated with SARS-CoV-2 spike protein. Overexpression with the wild-type ACE2 plasmid (which can be demethylated) significantly increased ACE2/spike interactions, whereas overexpression of ACE2_K31F, which cannot be de-methylated and mimics a hypermethylated status, significantly reduced ACE2/spike interactions, confirming the importance of lysine 31 PTM (Fig. 2f and Supplementary Fig. S2e). To address whether LSD1 demethylase activity is required for ACE2-spike protein interactions, Caco-2 cells were pre-treated with phenelzine and GSK prior to and following SARS-CoV-2 infection. Phenelzine, and to a greater extent GSK treatment, reduced spike protein expression at the cell surface of infected cells at 48 hpi (Fig. 2g and Supplementary Fig. S2f). Furthermore, while *LSD1*, *ACE2*, and *TMPRSS2* transcript remained unaltered in SARS-CoV-2-infected Caco-2 cells following phenelzine or GSK treatment, a limited type I interferon response was induced in Caco-2 cells following SARS-CoV-2 infection (Supplementary Fig. S2g). Compared to uninfected cells, the transcriptional response to infection including *IFNβ, RIG-1, MDA-5, ISG15*, and *OASL* was increased at 48 hpi, with *IFNβ* and *OASL* expression further increased following phenelzine treatment. mRNA levels of *RIG1, MDA5*, and *ISG15* remained unaltered by phenelzine or GSK treatment (Supplementary Fig. S2g).

RNA sequencing (RNA-seq) was performed to identify global gene expression programs impacted by LSD1 inhibition in SARS-CoV-2-infected Caco-2 cells. Principal component analysis (PCA) demonstrated good separation of samples according to their treatment type, and high similarity between biological replicates (Fig. 2h). Principal component (PC)1 separated phenelzine-treated samples from all other samples and accounted for 66% of the variance, whilst PC2 further separated GSK-treated samples from the control samples and accounted for 12% of the variance (Fig. 2h). Differential expression analysis revealed 1059 differentially expressed genes (DEGs) between phenelzine-treated vs. control samples and 73 DEGs between GSK treated vs. control samples, with 36 of these DEGs shared between the two contrasts (Supplementary Fig. S2h). Taken together, these results suggest that phenelzine and GSK regulate global host responses to prevent SARS-CoV-2 infection via different molecular mechanisms.

Next, we performed over-representation analysis using Reactome to investigate pathway enrichment of the DEGs from our two contrasts, phenelzine vs. control and GSK vs. control. Reactome enrichment analysis revealed that translation and MAPK pathways were enriched in phenelzine-treated samples, whilst GSK treatment downregulated genes enriched in the IFN pathway (Fig. 2i, j and Supplementary Fig. S2i). Furthermore, post-transcriptional protein phosphorylation and amino acid synthesis and interconversion (transamination) processes were significantly enriched in phenelzine-treated samples, which may have contributed to the decreased SARS-CoV-2 spike protein expression observed in infected cells (Fig. 2g). Taken together, LSD1 inhibition impacts key antiviral processes and proteins responsible for SARS-CoV-2 infection in host cells. The different amplitudes of effects of different LSD1 inhibitors may be attributable to their different modes of action, with phenelzine affecting catalytic, nuclear, and structural LSD1 functions. Regardless, LSD1 inhibition reduces spike protein interactions with ACE2, and LSD1-induced demethylation of ACE2 at the RBD may be required for sufficient engagement with the SARS-CoV-2 spike protein and subsequent viral internalization into the host cell.

To examine the effect of interactions between demethylated ACE2 lysine 31 and SARS-CoV-2 spike protein at the cell surface, we developed two novel ACE2 peptide inhibitors (ACE2-01 and ACE2-02) through structural analysis and modeling (Fig. 2b) of the target sequence, peptide length optimization, and alanine walking to identify critical residues. Overlapping the spike interaction region, ACE2-01 extends downstream past the lysine demethylation motif, while ACE2-02 extends and terminates at lysine 31 to facilitate studies of this critical residue (Fig. 2k). Both peptides are predicted to competitively block interactions between the spike protein and lysine 31, either by interfering with ACE2/spike interactions or by binding to the spike protein as a decoy. These peptides also competitively block enzymatic access to lysine 31 as a decoy interaction, interfering with lysine 31 demethylation by mimicking the ACE2/spike binding domain/lysine d-methylation motif, meaning that the LSD1 catalytic pocket or the RBD spike domain interacts with the peptide and not the target protein to prevent lysine 31 demethylation or spike–ACE2 interactions.

In comparison to untreated control cells, neither ACE2 peptide inhibitor altered cell proliferation up to 96 h of treatment (Fig. 2m). To assess the impact of ACE2-01 and ACE2-02 on SARS-CoV-2 replication, Caco-2 cells were infected with SARS-CoV-2 (MOI 1.0) and then treated with the peptide inhibitors for 48 h (Fig. 2l). Using qRT-PCR of both the culture supernatants and infected cells, ACE2-01 and ACE2-02 treatment significantly reduced infection by 6.6-fold and 4.6-fold, respectively, in cell culture supernatants at 48 hpi (Fig. 2n). Viral RNA also decreased by 3.3-fold and 2-fold in infected cells following ACE2-01 and ACE2-02 treatment at 48 hpi, respectively (Fig. 2n).

Next, infectious viral titers were quantified by median tissue culture infectious dose (TCID_50_) of supernatants from infected cells treated with ACE2-01 and ACE2-02, which further confirmed reductions in viral load by 4.5-fold and 3.2-fold, respectively (Fig. 2o). Furthermore, inhibition of SARS-CoV-2 infection was assessed using digital pathology to detect spike protein intensity (Fig. 2p and Supplementary Fig. S2j). Both inhibitors significantly reduced SARS-CoV-2 spike protein at the cell surface and intracellularly in infected cells (Fig. 2p and Supplementary Fig. S2j), with co-localization of spike and ACE2 also significantly reduced (Fig. 2p). Finally, a proximity ligation assay was used to assess the co-localization of ACE2 and spike protein at the surface of SARS-CoV-2-infected Caco-2 cells. GSK treatment significantly decreased interaction between ACE2 spike protein, and ACE2-01 and ACE2-02 peptide inhibitors further disrupted ACE2/spike complexes at the cell surface (Fig. 2q). This suggests that methylation of ACE2 via inhibition of LSD1 activity contributes to blocking access to ACE2 by the SARS-CoV-2 spike protein. Furthermore, competitively blocking access to the lysine 31 motif with peptide inhibitors inhibits spike protein access to ACE2. Taken together, these data demonstrate that interactions between the viral spike protein and the lysine 31 demethylation motif of ACE2 are important for SARS-CoV-2 replication, and our peptide inhibitors have antiviral activity by significantly reducing spike protein co-localization with ACE2.

### Co-existence of LSD1 and ACE2 in patient PBMCs is associated with COVID-19 disease severity

Antiviral lymphocytes, especially CD8^+^ T cells, are critical for viral control, and impaired adaptive immune responses are associated with disease progression. Lymphopenia and functional exhaustion of lymphocytes have been observed in many patients with COVID-19 infection^51–54^. In addition, human leukocytes^55^ and mouse T cells^56^ express cell surface ACE2, suggesting that SARS-CoV-2 can directly infect human T cells and subsequently lead to lymphopenia. Given that LSD1 regulates ACE2–spike interactions, we next investigated whether LSD1 complexes with ACE2 in lymphocytes in COVID-19 patients, with the aim of providing novel insights into antiviral immunity in COVID-19.

**Fig. 3:**
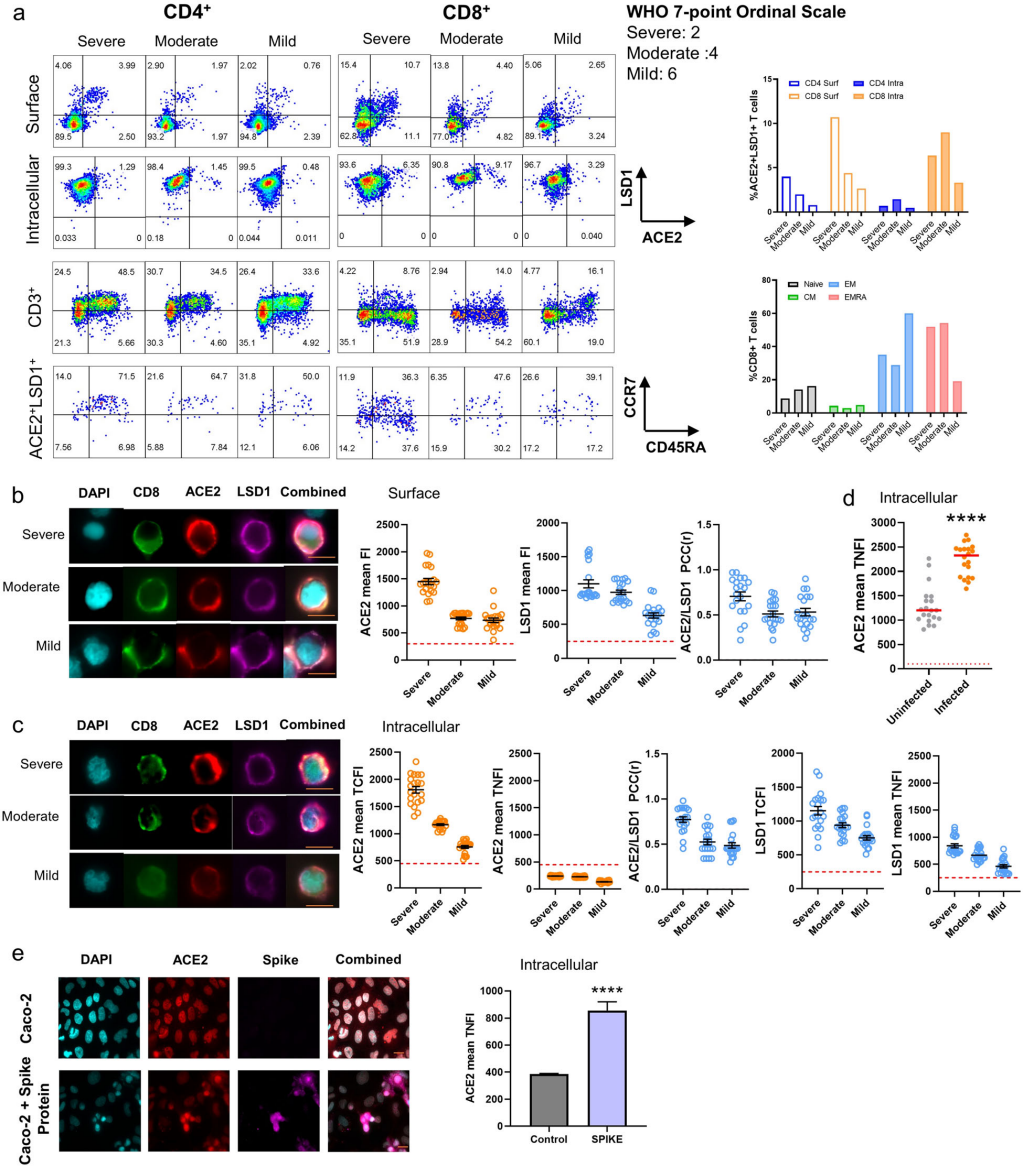
LSD1 co-localizes with ACE2 in SARS-CoV-2-infected primary cells. **a** FACS analysis of cell surface and intracellular expression of ACE2 and LSD1 and the cell subset composition of T cells in COVID-19 patient PBMCs (*n* = 3). The bar graph indicates the percentage ACE2^+^LSD1^+^ cells in the total Caco-2 population and each cell subset frequency of the total CD8^+^ T cells. **b** Representative image of PBMCs derived from COVID-19 patients using the ASI digital pathology system. Cells were not permeabilized (surface) for immunostaining for CD8, LSD1, and ACE2. DAPI (blue) was used to visualize nuclei. Scale bar, 12 μm (inset). Dot plot quantification of the fluorescence intensity (cell surface) of ACE2 and LSD1 in COVID-19 patient PBMCs (*n* = 3). >50 cells were analyzed for each patient sample. **c** Representative image of PBMCs derived from COVID-19 patients using the ASI digital pathology system. Cells were permeabilized (intracellular) for immunostaining for CD8, LSD1, and ACE2. DAPI (blue) was used to visualize nuclei. Scale bar, 12 μm (inset). Dot plot quantification of the fluorescence intensity (cytoplasmic and nuclear) of ACE2 and LSD1 in COVID-19 patient PBMCs (*n* = 3). >50 cells were analyzed for each patient sample. **d** Dot plot quantification of the fluorescence intensity (nuclear) of ACE2 in uninfected or SARS-CoV-2-infected Caco-2 cells. >50 cells were analyzed for each group. Mann–Whitney-test: ^∗∗∗∗^*P* < 0.0001 denote significant differences. **e** The ASI digital pathology system was used to analyze ACE2 expression in Caco-2 cells treated with the SARS-CoV-2 spike protein. Dot plots displays the nuclear fluorescence intensity in Caco-2 cells for ACE2 in control cells or cells positive for the spike protein. *n* > 1000 cells counted per group. Data represent mean ± SEM. Mann–Whitney test. ^∗∗∗∗^*P* < 0.0001 denotes significant differences. n.s. non-significant.

To assess the immunophenotype of COVID-19 patients, we collected peripheral blood mononuclear cells (PBMCs) from hospitalized patients with mild, moderate, and severe disease according to the WHO seven-point ordinal scale^57^. Flow cytometry analysis with an eight-color immunophenotyping panel showed that both primary CD4^+^ and CD8^+^ T cells derived from a patient with severe COVID-19 had the highest cell surface LSD1–ACE2 co-expression, followed by lower expression in moderate disease and even lower expression in mild disease (Fig. 3a). CD8^+^ T cells expressed much higher levels of surface ACE2 and LSD1 than CD4^+^ T cells. Only CD8^+^ T cells, but not CD4^+^ T cells, expressed intracellular ACE2.

We next profiled the composition of T cell subsets in COVID-19 patients. CD3^+^ T cells were less frequent in both moderate and severe COVID-19 patients than mild disease patients (data not shown). Importantly, the frequency of naïve CD8^+^ T cells (CCR7^+^CD45RA^+^) was two-fold lower in the patient with severe disease compared to the patient with mild disease, while a remarkably higher frequency of terminally differentiated T_EMRA_ (CCR7^−^CD45RA^+^) CD8^+^ T cells was observed in both moderate (54.2%) and severe (51.9%) COVID-19 compared to in mild COVID-19 (19.0%). Notably, there was no significant difference in CD4^+^ T cell subset composition according to disease severity (Fig. 3a). Consistent with Urra, Cabrera, Porras, and Ródenas 53, SARS-CoV-2 infection preferentially affected primary CD8^+^ T cells, not CD4^+^ T cells, and ACE2 and LSD1 co-expression in CD8^+^ T cells may be prognostic. Moreover, similar ACE2 and LSD1 co-expression was observed at the cell surface and intracellularly in CD3^−^ cells, with the highest levels in severe disease (Supplementary Fig. S3). Finally, the majority of ACE2^+^LSD1^+^ cells were naïve T cells, suggesting that SARS-CoV-2 may preferentially infect naïve T cells. We therefore hypothesize that SARS-CoV-2 might be able to directly infect primary T cells and induce T cell apoptosis, eventually compromising the antiviral immune response and contributing to disease progression.

Next, we examined cell surface, cytoplasmic, and nuclear ACE2 and LSD1 expression in COVID-19 patients. The expression and co-localization of LSD1 and ACE2 at the cell surface and intracellularly in CD8^+^ T cells were also associated with patient disease severity (Fig. 3b, c). Notably, however, nuclear ACE2 was undetectable (Fig. 3c). In contrast, nuclear ACE2 was significantly induced in Caco-2 cells after SARS-CoV-2 infection (Fig. 3d). We next treated Caco-2 cells with recombinant SARS-CoV-2 spike protein S1 (residues 14–680 of the S1 subunit) for 24 h, which also induced nuclear translocation of ACE2 in Caco-2 cells (Fig. 3e). Therefore, nuclear ACE2 may play an important role in regulating SARS-CoV-2 replication in host cells, but SARS-CoV-2 may not be able to replicate in infected T cells, similar to MERS-infected T lymphocytes^58^.

### Nuclear ACE2 is shuttled to the nucleus via LSD1 demethylation-dependent interactions with the shuttling protein importin alpha

**Fig. 4:**
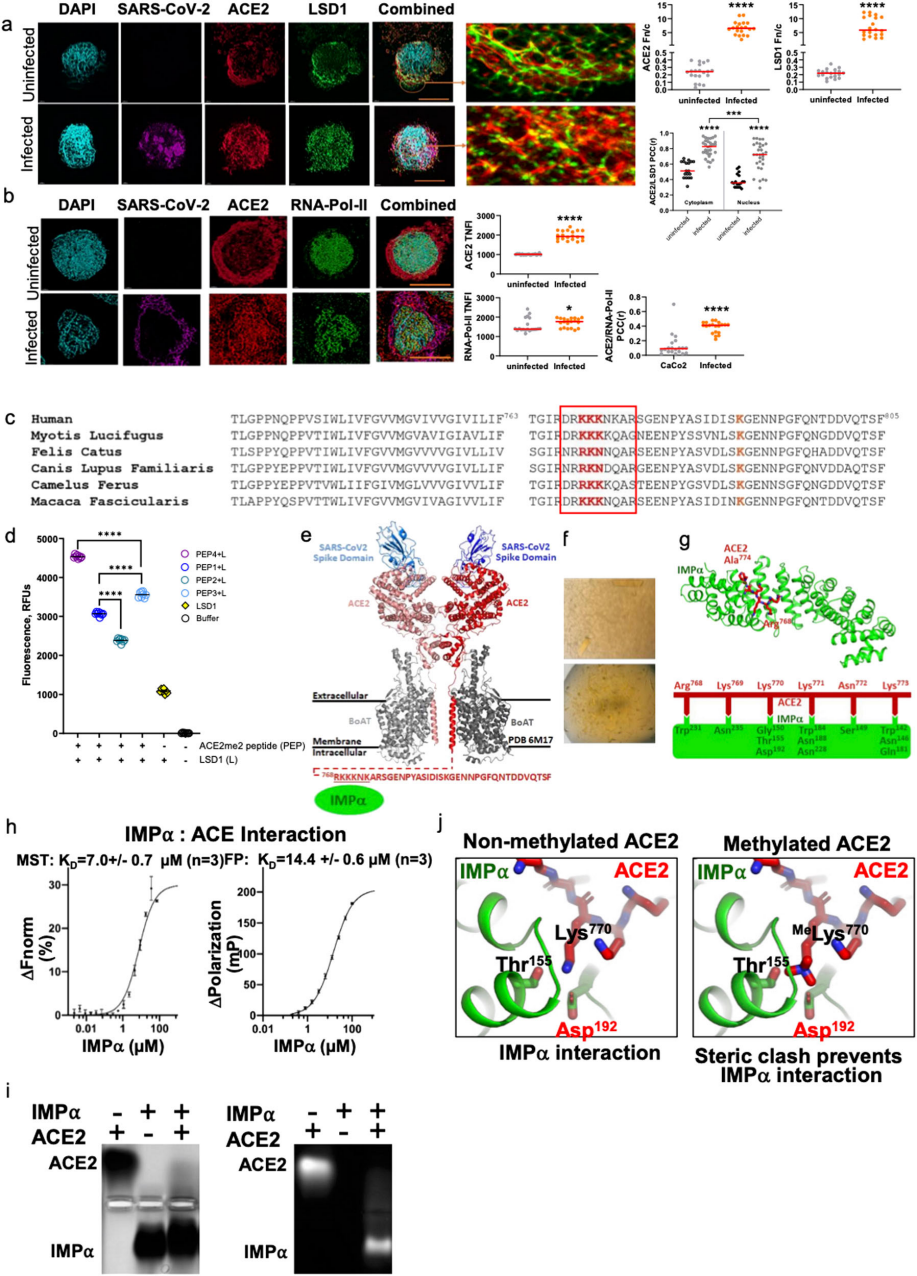
LSD1 directly interacts with ACE2. **a** Representative image of uninfected or SARS-CoV-2-infected Caco-2 cells using the Andor WD Revolution Inverted Spinning Disk microscopy system. Cells were permeabilized (intracellular) for immunostaining for ACE2, LSD1, and SARS-CoV-2 N (nucleocapsid). DAPI (blue) was used to visualize nuclei. Scale bar, 12 μm (inset). The ratio of nuclear to cytoplasmic staining (Fn/c) was also calculated for LSD1 and ACE2: >1 indicates nuclear bias, whereas <1 indicates cytoplasmic bias. The PCC was calculated to assess colocalization in Caco-2 cells with/without infection in the cytoplasmic or nuclear compartments (*n* = 20 cells analyzed). Data are mean ± SEM. Mann–Whitney test: ^∗∗∗^*P* < 0.001, ^∗∗∗∗^*P* < 0.0001 denote significant differences. **b** Representative image of uninfected or SARS-CoV-2-infected Caco-2 cells using the Andor WD Revolution Inverted Spinning Disk microscopy system. Cells were permeabilized (intracellular) for immunostaining for ACE2, RNA Pol-II, and SARS-CoV-2 N (nucleocapsid). DAPI (blue) was used to visualize nuclei. Scale bar, 12 μm (inset). Dot plot quantification of the fluorescence intensity of ACE2 and RNA Pol-II in uninfected or SARS-CoV-2-infected Caco-2 cells. >50 cells were analyzed for each group. The PCC was calculated for colocalization (*n* = 20 cells were analyzed). Mann–Whitney test: ^∗^*P* < 0.05, ^∗∗∗∗^*P* < 0.0001 denote significant differences. **c** Representative conservation of the ACE2 c-terminal sequence across multiple species. An NLS (red box) is identified with three lysines highly likely to be subject to acetylation/demethylation (red). This region may be involved in protein stabilization as there is also a nearby lysine (in orange) highly likely to be ubiquitinated for proteasomal degradation. **d** ACE2me2 peptide substrate demethylation by LSD1. LSD1 activity was measured by demethylation assay with ACE2me2 peptide of PEP1, PEP2, PEP3, or PEP4. Fluorescent was measured at 40 s intervals on a Synergy H4 multi-mode plate reader. The background rate of ACE2me2 peptide was subtracted in corresponding samples. Statistical significance was calculated using one-way ANOVA, ^∗∗∗∗^*P* < 0.0001 denote significant differences. **e** The C-terminal, cytoplasmic domain of ACE2 contains a putative NLS. This region was unresolved in the recently determined structure (PDB 6M17). Shown is a cartoon representation of the ACE2 structure and the sequence of the unresolved C-terminal domain of ACE2. Underlined residues represent the putative NLS. **f** Crystals of importin-α bound to FITC-Ahx tagged ACE2 used in diffraction and structure determination. **g** Structure of importin-α bound to ACE2. Importin-α, shown in cartoon mode (green), bound to ACE2, shown in stick mode (red). Interacting residues are schematically presented with the same color scheme as above. Zoom inset shows that Lys770 forms a critical interaction with the P2 site of importin-α, and methylation of this site would form steric clashes. **h** Microscale thermophoresis (left) and fluorescence polarization (right) was used to assess the strength of binding between importin-α and ACE2. Each experiment was carried out with an *n* = 3, with the KD shown representing the mean and standard deviation. **i** The electrophoresis mobility shift assay was carried out to confirm the interaction between IMPα and ACE2 via the C-terminal domain. ACE2 C-terminal domain is FITC labeled. Left panel is Coomassie stained, right panel is visualized by UV. **j** Depicts 3D model of ACE2 interaction with importin-α via the NLS in the C-terminal region at Lysine 769, 770, and 771. This model demonstrates that methylation of these lysines would cause steric hindrance and prevent interaction, indicating that ACE2 methylation and non-methylation are important for the importin-α interaction.

Given that nuclear ACE2 may play an important role in SARS-CoV-2 infection, super-resolution imaging of infected or uninfected Caco-2 cells was performed to examine intracellular ACE2 and LSD1 expression. LSD1 and ACE2 colocalized in the cytoplasm, but there was also a significant increase in nuclear ACE2/LSD1 colocalization upon SARS-CoV-2 infection (Fig. 4a). The nuclear to cytoplasmic staining ratio (Fn/c) was also calculated using super-resolution imaging data of LSD1 and ACE2 in infected vs. uninfected Caco-2 cells. Infection with SARS-CoV-2 significantly increased the Fn/c of both LSD1 and ACE2, clearly indicating a significant nuclear bias for LSD1 and ACE2 upon infection (Fig. 4a).

Next, we examined potential partners of nuclear ACE2 using super-resolution imaging. First, ACE2-RNA-Pol-II colocalization was assessed within nuclei of SARS-CoV-2-infected Caco-2 cells. ACE2 and RNA-Pol-II were significantly upregulated following infection and, additionally, ACE2-RNA-Pol-II colocalization increased in infected cells, suggesting that nuclear ACE2 is associated with active transcriptional processes during SARS-CoV-2 infection (Fig. 4b).

We also explored potential mechanisms of LSD1-mediated ACE2 nuclear translocation in silico. In addition to the N-terminal demethylation domain identified above, we also identified a high score (prediction cut off of 0.7 out of 1), highly conserved demethylation domain within the cytoplasmic tail of ACE2 (Fig. 4c) harboring a high-affinity, highly conserved nuclear localization sequence (NLS) using both the previously mentioned bioinformatics tool from Wen et al.^34^ to identify the de-methylation domain (TGIRDRKKKNKRS) and the NLS prediction tool NLStradamus^59^ to identify the NLS within this motif (RKKKNK).

Moreover, to assess whether LSD1 regulates the demethylation of ACE2 in the NLS domain, ACE2 peptides di-methylated at either lysine 769(PEP1), 770 (PEP2), 771 (PEP3) or double di-methylated at lysine 769 and 771 (PEP4) within the NLS motif were constructed and used as substrates for in vitro LSD1 activity assays. Recombinant LSD1 showed the greatest demethylation of the double di-methylated ACE2 peptide (PEP4) followed by PEP3, PEP2, and PEP1 (Fig. 4d and Supplementary Table S1).

We hypothesized that ACE2 demethylation by LSD1 surrounding the NLS or targeting the triple lysine motif comprising the NLS may influence interactions between ACE2 and the importin-α shuttling protein to translocate into the nucleus following SARS-CoV-2 infection (Fig. 4e). To examine the structural basis for any interaction between ACE2 and importin-α, we crystallized the cytoplasmic tail of ACE2 with importin-α (Fig. 4f). The structure, resolved at 2.2 Å (Table 1), revealed ACE2 lysine residues forming important interactions with importin-α (Fig. 4g), and a direct, micromolar interaction was confirmed in both MST and FP assays (Fig. 4h). The interaction between importin-α and ACE2 c-terminal domain was further confirmed using an electrophoretic mobility shift assay (Fig. 4i). Examination of the interaction interface suggested that lysine methylation would cause steric clashes and severely impact the interaction between ACE2 and importin-α (Fig. 4j) and its capacity to shuttle cargo to the nucleus (Supplementary Table S2).

**Table 1:** Data collection and refinement statistics.

Data collection and processing	IMPα:ACE2
Wavelength (Å)	0.9537
Resolution range (Å)	29.98–2.20 (2.27–2.20)
Space group	P 21 21 21
Unit cell (Å, ^o^)	78.94 89.95 100.09 90 90 90
Total reflections	252,620 (21,940)
Unique reflections	36,873 (3125)
Multiplicity	6.9 (7.0)
Completeness (%)	99.9 (99.8)
Mean I/sigma(I)	15.9 (1.8)
Wilson B-factor Å^2^	33.4
R-merge	0.095 (1.095)
R-pim	0.054 (0.649)
CC1/2	0.999 (0.807)
*Refinement*	
Number of reflections	36,805
Number of R-free reflections	1803
R-work (%)	0.1863
R-free (%)	0.2099
RMS (bonds)	0.004
RMS (angles)	0.67
*Ramachandran plot*	
Favored (%)	98.83
Allowed (%)	1.17
Outliers (%)	0
Average B-factor Å^2^	48.08
Clash score	2.92
PDB accession code	7JVO

Statistics for the highest-resolution shell are shown in parentheses.

In summary, LSD1 can de-methylate ACE2 in the NLS located within the cytoplasmic tail to regulate interactions between ACE2 and importin-α to control the nuclear localization of ACE2.

### A novel ACE2 peptide inhibitor

**Fig. 5:**
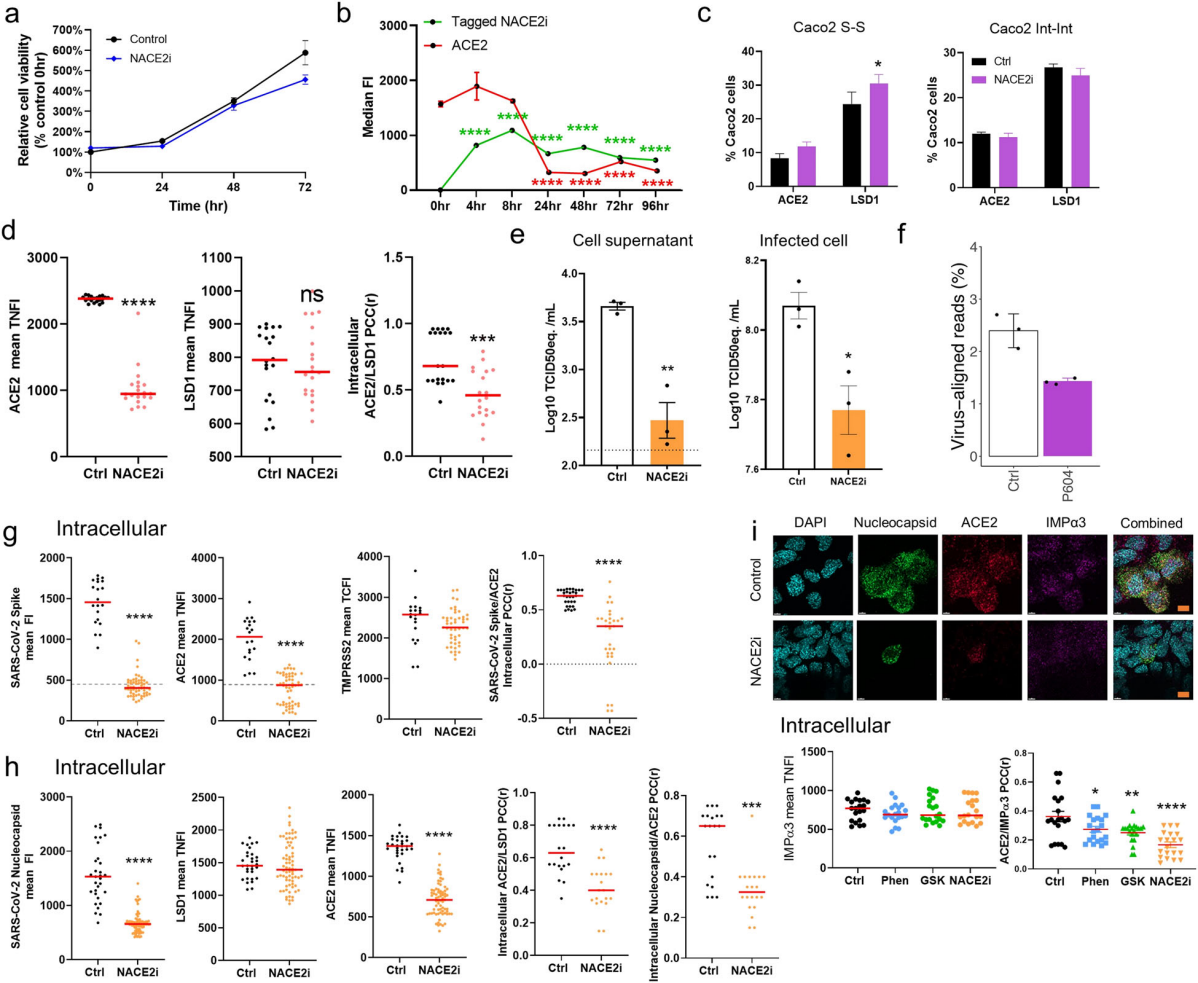
A novel ACE2 peptide inhibitor. **a** Cell proliferation analysis of Caco-2 control (depicted in Fig. 3) and NACE2i (50 μM)-treated cells over a 72 h period. Proliferation was analyzed using WST-1 reagent and absorbance read after 2 h incubation. The graph depicts relative cell proliferation from triplicates expressed as a percentage of control cells (untreated, 0 h). Statistical significance was calculated using one-way ANOVA at each time point. **b** The ASI Digital pathology system was used to analyze ACE2 expression over time in Caco-2 cells treated with NACE2i (with FAM5 TAG). Dot plots displays the nuclear fluorescence intensity in Caco-2 cells for ACE2 and FAM5 tag as well as the % population of ACE2-expressing cells, >20 cells counted per group. Data represent mean ± SEM. Mann–Whitney test. ^∗∗∗∗^*P* < 0.0001 denote significant differences. **c** FACS analysis of cell surface and intracellular expression of ACE2 and LSD1 in Caco-2 cells treated with/without NACE2i for 48 h. The bar graph indicates the percentage ACE2^+^ or LSD1^+^ cells of the total Caco-2 population. Data are mean ± SEM (*n* = 3). Mann–Whitney test. ^∗^*P* < 0.05 denote significant differences. **d** Dot plot quantification of the fluorescence intensity (intracellular) of ACE2 and LSD1 in Caco-2 cells with NACE2i treatment. >50 cells were analyzed for each group. The PCC was calculated for colocalization (*n* = 20 cells were analyzed). Mann–Whitney-test: ^∗∗∗^*P* < 0.001, ^∗∗∗∗^*P* < 0.0001 denote significant differences. **e** qRT-PCR analysis to detect replicates of SARS-CoV-2 RNA in Caco-2 culture supernatants and infected cells at the indicated time points post-infection. The quantity of viral genome is expressed as TCID_50_ equivalents/mL. Data represent mean ± SEM, *n* = 3. One-way ANOVA, ^∗^*P* < 0.05, ^∗∗^*P* < 0.01 denote significant differences. **f** SARS-CoV-2 levels in infected Caco-2 cells. The percentage of virus-aligned reads (over total reads) is indicated for each sample. Error bars represent standard deviation from three independent biological replicates. **g** Dot plot quantification of the fluorescence intensity (intracellular) of SARS-CoV-2 spike, ACE2, LSD1, and TMPRSS2 in SARS-CoV-2-infected Caco-2 cells with phenelzine, GSK, or NACE2i treatment. >50 cells were analyzed for each group. The PCC was calculated to assess colocalization (*n* = 20 cells were analyzed). Data are mean ± SEM. Mann–Whitney-test: ^∗∗∗∗^*P* < 0.0001 denote significant differences. **h** Dot plot quantification of the fluorescence intensity (intracellular) of SARS-CoV-2 nucleocapsid, ACE2, and LSD1 in SARS-CoV-2-infected Caco-2 cells with phenelzine, GSK, or NACE2i treatment. >50 cells were analyzed for each group. The PCC was calculated to assess colocalization (*n* = 20 cells were analyzed). Data are mean ± SEM. Mann–Whitney-test: ^∗∗∗^*P* < 0.001, ^∗∗∗∗^*P* < 0.0001 denote significant differences. **i** Representative image of uninfected or SARS-CoV-2 infected Caco-2 cells using the Andor WD Revolution Inverted Spinning Disk microscopy system. SARS-CoV-2-infected Caco-2 cells with phenelzine, GSK, or NACE2i treatment, followed by permeabilization (intracellular) for immunostaining for SARS-CoV-2 nucleocapsid, ACE2, and IMPα3. DAPI (blue) was used to visualize nuclei. Scale bar, 12 mm (inset). Dot plot quantification of the fluorescence intensity of IMPα3 in SARS-CoV-2-infected Caco-2 cells. >50 cells were analyzed for each group. The PCC was calculated to assess colocalization (*n* = 20 cells were analyzed). Data are mean ± SEM. Mann–Whitney test: ^∗^*P* < 0.05, ^∗∗^*P* < 0.01, ^∗∗∗∗^*P* < 0.0001 denote significant differences.

To address the importance of nuclear ACE2 in SARS-CoV-2 infection, we developed a cell permeable peptide (P604/NACE2i) spanning the ACE2-importin-LSD1 demethylation domain (Supplementary Fig. S4a) optimized according to peptide length and alanine walking to identify critical residues. The NACE2i peptide did not alter Caco-2 cell proliferation (Fig. 5a). Next, we profiled the stability of FAM5-tagged NACE2i peptide over 96 h. With a single peptide treatment, the peptide was stable over time and significantly inhibited nuclear ACE2 expression after 8 h and up to 96 h (Fig. 5b and Supplementary Fig. S4b). Examining ACE2 and LSD1 expression at both the cell surface and intracellularly in Caco-2 cells, compared to the control group, NACE2i did not alter the frequency of ACE2^+^ and LSD1^+^ Caco-2 cells (Fig. 5c and Supplementary Fig. S4c).

To investigate the effect of NACE2i treatment on ACE2 and LSD1 expression, nuclear ACE2 and LSD1 expression and colocalization were measured. NACE2i treatment significantly reduced nuclear ACE2 mFI but had no effect on intracellular LSD1 (Fig. 5d). Intracellular ACE2 and LSD1 colocalization was also significantly disrupted by peptide inhibition (Fig. 5d).

To determine whether our treatments inhibited SARS-CoV-2 infection in Caco-2 cells, viral RNA was quantified in supernatants 0, 24, and 48 hpi (Fig. 5e). Compared to control cells, which showed significantly increased SARS-CoV-2 RNA replication over 48 hpi, NACE2i treatment significantly decreased SARS-CoV-2 replication in infected Caco-2 cells compared to control cells at 48 hpi. Lastly, RNA sequencing was performed on the poly(A) RNA isolated from infected cells to estimate the % viral load. Consistent with qRT-PCR data, GSK (1% total reads) and P604/NACE2i (1.5% total reads) treatment reduced viral load compared to control cells (2.5% total reads) (Fig. 5f).

We next used untagged NACE2i to examine its effects on nuclear ACE2 and expression of the SARS-CoV-2 spike and nucleocapsid proteins. Although *LSD1*, *ACE2*, and *TMPRSS2* expression was comparable in SARS-CoV-2-infected Caco-2 cells following treatment with NACE2i (Supplementary Fig. S4d), peptide treatment significantly destabilized nuclear ACE2 and also abrogated both spike and nucleocapsid protein expression (Fig. 5g, h). Furthermore, NACE2i significantly inhibited colocalization of ACE2:spike and ACE2:LSD1 and destabilized nuclear ACE2 complexes compared to control cells (Fig. 5g, h).

Given the vital role played by importin-α in shuttling ACE2 into the nucleus (Fig. 4), we next examined the dynamics of LSD1 inhibition (phenelzine and GSK), and our novel NACE2i on the dynamics of ACE2 and IMPα3 interactions and expression of SARS-CoV-2 nucleocapsid protein using super-resolution imaging. Treatment with GSK or phenelzine had no effect on IMPα3 nuclear expression and minimal effect on the overall co-localization of ACE2 and IMPα3. However, NACE2i strongly inhibited the colocalization of ACE2 and IMPα3 (Fig. 5i).

## Discussion

**Fig. 6:**
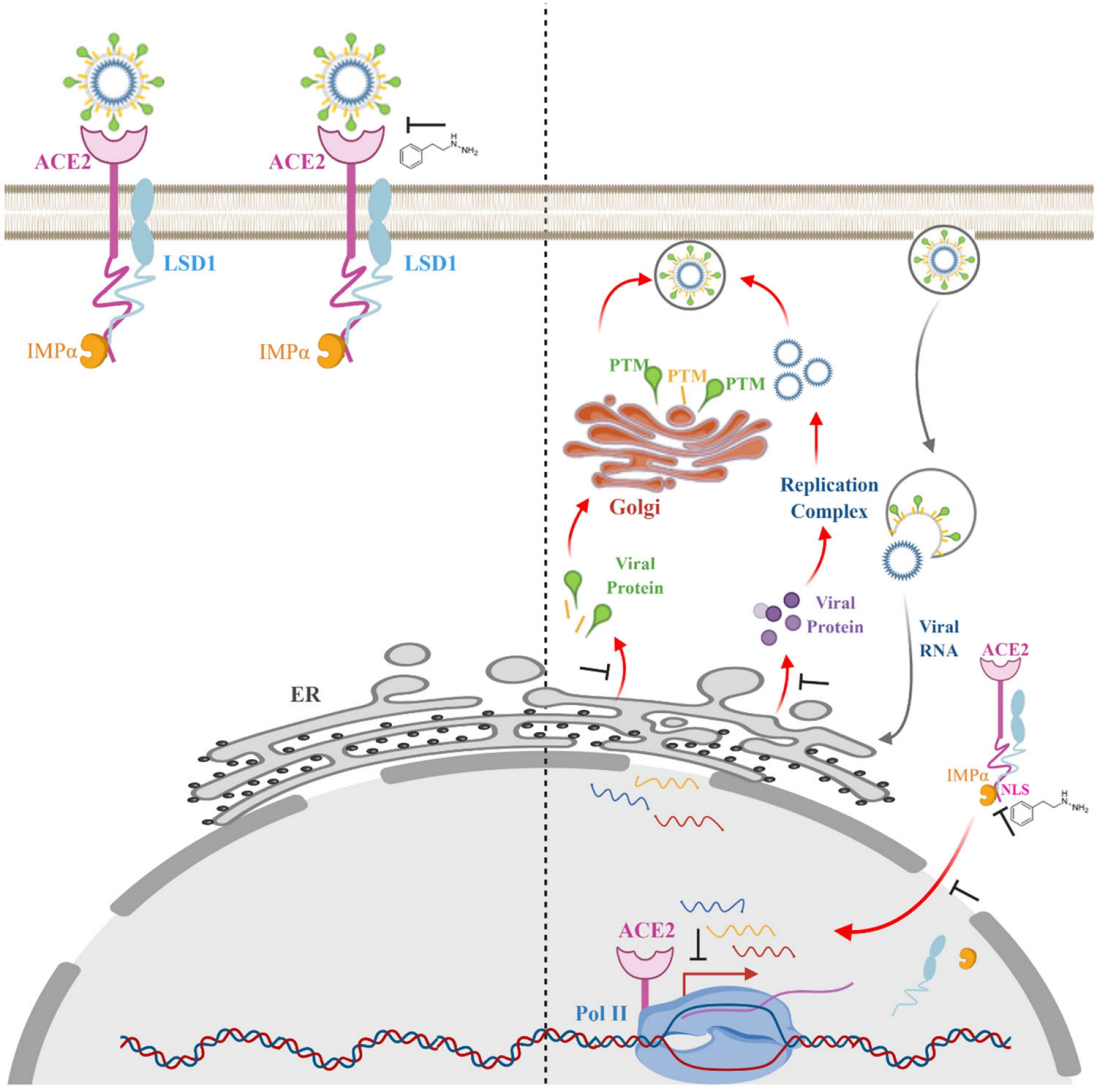
A summary model of SARS-CoV-2/ACE2/LSD1 interactions. Our data support a model in which: (Left) at the cell surface, SARS-CoV-2 entry relies on LSD1-dependent demethylation of ACE2, which is required for ACE2/SARS-CoV-2 spike binding. LSD1 inhibition therefore reduces viral entry via ACE2, especially LSD1 catalytic inhibitors such as GSK. In addition, our novel ACE2 peptide inhibitors, which compete with binding to SARS-CoV-2 spike protein, also block viral replication. (Right) Intracellularly, NACE2i blocks LSD1/ACE2 binding at cytoplasmic tail, and this change in protein–protein interactions might consequently inhibit LSD1’s demethylation activity. IMPα binds to the cytoplasmic tail of non-methylated ACE2 to mediate nuclear translocation and Pol II association to regulate gene expression. Therefore, NACE2i blocking IMPα/ACE2 binding impact ACE2-Pol II nuclear co-localization, which is required for SARS-CoV-2-related transcription, to reduce viral replication.

In this study, our data and model (Fig. 6) suggest two potential inhibition strategies for COVID-19 infection: (1) blocking SARS-CoV-2 viral replication by either inhibiting LSD1 demethylation using re-purposed drugs (phenelzine and GSK) or by competing with binding to SARS-CoV-2 spike protein using novel ACE2 peptide inhibitors; or (2) preventing ACE2 nuclear translocation by blocking IMPα3 binding to the ACE2 cytoplasmic tail, subsequently diminishing the ACE2-Pol II co-localization required for SARS-CoV-2-related active transcription. This work extends recent findings highlighting the importance of targeting the importin nuclear transport machinery to effectively treat SAR-CoV-2 infection.

Here we characterized SARS-CoV-2 spike binding to ACE2 and the role of epigenetic eraser enzyme LSD1 in this interaction. LSD1 colocalizes with ACE2 at the cell surface to maintain demethylated lysine 31 in the SARS-CoV-2 spike RBD to promote the interaction between SARS-CoV-2 spike protein and ACE2. We identified a methylation/demethylation motif at lysine 31 on ACE2. ACE2 lysine 31 binds to SARS-CoV-2 glutamine 493 in the spike RBD. Our data demonstrate that LSD1 colocalizes with ACE2 both at the cell surface and intracellularly in SARS-CoV-2-susceptible cells. We reveal for the first time that LSD1 has a novel cell surface role in the context of SARS-CoV-2 infection, promoting efficient ACE2–SARS-CoV-2 spike interactions via demethylation of ACE2 lysine 31. This novel role for LSD1 at the cell surface is consistent with other findings showing a key role for enzymatic activity at the cell surface^60,61^.

Of note, although LSD1 inhibition with re-purposed drugs such as phenelzine and GSK negligibly reduced viral RNA levels in culture supernatants from infected Caco-2 cells, both treatments significantly reduced the co-localization of ACE2 and SARS-CoV-2 spike protein and decreased SARS-CoV-2 spike protein expression in infected Caco-2 cells at 48 hpi. Moreover, GSK treatment showed the greatest inhibition of viral RNA in infected cells at 48 hpi in global RNA-seq analysis. The viral RNA detected in culture supernatants normally indicates newly produced virus particles released from infected cells, so despite viral RNA and spike protein inhibition in infected cells by both treatments, these effects may take time to develop. Therefore, ACE2 lysine 31 demethylation may be a novel therapeutic target for COVID-19, and re-purposed phenelzine and GSK might be useful for inhibiting SARS-CoV-2 infection. However, longer periods of post-infection treatment with infectious virus titer monitoring need to be examined to confirm these findings.

Epigenetic drugs are promising antiviral treatments capable of modifying epigenetic tags and re-programming host and viral genomes. In addition to the predicted block of SARS-CoV-2 entry through LSD1 inhibition, phenelzine also altered host transcription of IFN, MAPK, and translation-related pathways, which may further benefit the control of SARS-CoV-2 infection. Type I IFNs have been shown to be essential in the control of SARS-CoV-2 infection^62^. TLR3 and IRF7 deficiencies have been reported to be associated with impaired type I IFN production and underpin life-threatening COVID-19 pneumonia in humans. Phenelzine has previously been shown to epigenetically restore T cell responses in human breast cancer^63^. Unsurprisingly, we found that phenelzine treatment regulated IFN pathways in SARS-CoV-2-infected Caco-2 cells, especially upregulation of *IRF7* gene expression. In addition, COVID-19 patients with severe disease have been shown to suffer from a rapid and excessive production of harmful pro-inflammatory cytokines (IL6, IL1-β, and TNF-α), or “cytokine storm”, triggered by the innate immune response against the virus^64–66^. Phenelzine acts as an antidepressant through monoamine oxidase inhibition (MAOi), and since antidepressants affect circulating cytokine and chemokine levels in vitro and in individuals with major depressive disorder (MDD)^67^ and anti-depressants also have neurotransmitter-independent effects on pro-inflammatory cytokine production^68^, we propose that the MAOi antidepressant effects of phenelzine may also promote host antiviral responses. A recent study of the global SARS-CoV-2 phosphorylation landscape indicated that SARS-CoV-2 infection upregulates p38/MAPK signaling, and several pharmacological inhibitors of p38/MAPK activity suppressed inflammatory cytokine production and SARS-CoV-2 replication^69^. Of note, our global RNA-seq analysis revealed that phenelzine treatment also regulates MAPK signaling-related pathways. Particularly, several dual-specificity phosphatases (DUSPs) such as *DUSP1, DUSP6*, and *DUPS8*, which are known to inhibit MAPKs and p38 by dephosphorylation, were highly upregulated by phenelzine in SARS-CoV-2-infected Caco-2 cells. Therefore, we hypothesize that the antidepressant phenelzine can be repurposed to target LSD1 to control viral infection, exuberant inflammation, and dampen the “cytokine storm” in COVID-19 patients. Moreover, a recent study of virus–human protein–protein interactions indicated that SARS-CoV-2 can hijack the host translational machinery to prioritize the production of viral proteins for their replication and assembly^70^. Several translation-related pathways were identified in phenelzine-treated cells upon SARS-CoV-2 infection, suggesting that phenelzine might reduce viral protein production by modulating post-translational protein phosphorylation and amino acid synthesis and interconversion (transamination). Therefore, phenelzine may not only impair viral protein synthesis but also enhance the host immune responses to control SARS-CoV-2 infection by inducing IFN I responses and suppressing inflammation.

Although ACE2 has been identified as a key receptor for SARS-CoV-2 entry into host cells, it also has been reported to protect the lung from injury^71,72^. Human recombinant soluble ACE2 (hrsACE2) significantly reduced viral load and inhibited the early stages of SARS-CoV-2 infection without loss of ACE2 expression. Consistent with this, our novel ACE2 peptides (ACE2-01 and ACE2-02) also potently inhibited SARS-CoV-2 infection in the absence of ACE2 expression changes by specifically preventing demethylation of ACE2 lysine 31 and competing for the binding of SARS-CoV-2 spike protein to host cell ACE2. Given that phenelzine may enhance antiviral immunity, the combination of phenelzine and ACE2 peptide inhibitor may have therapeutic potential to control COVID-19 infection, especially in patients with inborn errors of type I IFN immunity and severe COVID-19 disease with cytokine storm. Future clinical studies will be needed to formally address these possibilities.

COVID-19 patients with severe disease have fewer circulating functional T cells and NK cells and greater numbers of dysfunctional, exhausted CD8^+^ T cells^54,73^, suggesting that cellular immunodeficiency contributes to disease progression and clinical deterioration. Moreover, it has been reported that MERS-CoV can directly infect primary human T cells (both CD4^+^ and CD8^+^) and induce T cell apoptosis^58^. Thus, it is critical to screen for different T cell signatures upon COVID-19 infection to evaluate viral load and develop clinical applications for viral clearance. Strikingly, with increasing disease severity, ACE2 and LSD1 co-expression markedly increased in CD8^+^ T cells and CD3^−^ cells at both cell surface and intracellularly. By contrast, there was a minimal impact of SARS-CoV-2 on CD4^+^ T cells with respect to ACE2/LSD1 co-expression and cell subset composition, supporting previous findings that CD8^+^ T cells, but not CD4^+^ T cells, significantly decline in intensive care unit (ICU) patients compared to non-ICU patients^53^. Furthermore, ACE2^+^LSD1^+^ cells were predominately observed in the naïve CD8^+^ T cell population, together with a reduction in the naïve cell compartment, and expansion of T_EMRA_ subsets with increasing disease severity. We hypothesize that SARS-CoV-2 can directly attack naïve CD8^+^ T cells to deplete them by inducing apoptosis or driving naïve cell differentiation to terminally differentiated CD8^+^ T_EMRA_ cells. Compartment shrinkage of naïve CD8 T cells is the hallmark of immune aging in the elderly, who have poor immune responses to vaccination and fail to maintain a diverse T cell repertoire^74^. Indeed, elderly, immunocompromised humans are more susceptible to SARS-CoV-2 infection and poor clinical outcomes. The further depletion of naïve T cells in the elderly upon COVID-19 infection may contribute to more severe lymphopenia over time and inadequate CD8^+^ T cell responses against SARS-CoV-2.

ACE2 was initially identified as protective monocarboxypeptidase highly expressed in the human heart and kidneys^75,76^. Our findings show that ACE2 belongs to a growing number of plasma membrane proteins, that also have nuclear roles regulated via PTMs catalyzed by epigenetic enzymes that regulate transcription. Here, we show that demethylation of ACE2 in the cytoplasmic tail is required for interaction with the importin-α complex and nuclear translocation. This is similar to recent findings showing that the immune checkpoint protein PD-L1, a major immunotherapy target, also translocates to the nucleus to control transcription in an importin-α-dependent manner via PTM regulation of its cytoplasmic tail through the non-histone role of epigenetic enzymes^77^. Furthermore, other important receptors such as EGFR also have nuclear functions mediated by PTMs catalyzed by epigenetic enzymes that allow nuclear translocation^78^. Consistent with our findings, Gwathmey et al.^79^ reported nuclear ACE2 expression in renal proximal tubule cells. Similarly, nuclear ACE2 was also detected in Caco-2 cells, which increased upon SARS-CoV-2 infection. However, ACE2 was undetectable in the nuclei of CD8^+^ T cells, suggesting that nuclear ACE2 may be specific to epithelial cell phenotypes in COVID-19, where it may be a useful therapeutic target. As expected, our novel NACE2i, which prevented the nuclear translocation of ACE2 by disrupting the ACE2-importin-LSD1 complex, inhibited SARS-CoV-2 infection in Caco-2 cells. Notably, NACE2i showed no cytotoxicity nor changes in ACE2 expression in Caco-2 cells. We propose that intracellular ACE2 in NACE2i-treated cells retains its enzymatic capacity to convert ANG II to ANG-(1–7) on nuclear membranes to attenuate ANG II-dependent generation of oxidative stress while inhibiting viral replication by blocking its nuclear translocation to colocalize with Pol II, which may be required for active transcription in SARS-CoV-2 infection.

Taken together, our data show for the first time the interplay between epigenetic enzymes, the importin nuclear transport pathway, and nuclear ACE2 in the context of SARS-CoV-2 infection. Our data support the notion that targeting the nuclear axis hijacked by SARS-CoV-2 to re-direct host cell responses is critical for successfully therapeutic targeting in COVID-19. We also show here for the first time a novel cell surface role for LSD1 in the regulation of ACE2, which offers a new avenue for therapeutic targeting. Future studies utilizing LSD1 mutants will be required to probe these mechanisms underpinning cell surface localization of LSD1. These findings will be validated in a larger sample of primary cells and in authentic animal models of SARS-CoV-2.

## Materials and methods

### Cell culture

Caco-2 (human epithelial colorectal adenocarcinoma) and MRC-5 (human lung fibroblast) cell lines were cultured in Dulbecco’s Modified Eagle’s Medium (Gibco, Life Technologies, Carlsbad, CA) with 10% fetal bovine serum (FBS), 1× l-glutamine (Gibco), and 1× penicillin–streptomycin–neomycin solution (PSN) (Sigma Aldrich, St. Louis, MO). Vero E6 (C1008, ECACC, Wiltshire, England; Sigma Aldridge) cells were cultured in medium comprising RPMI 1640 (Gibco) supplemented with 10% fetal calf serum (FCS), penicillin (100 IU/mL)/streptomycin (100 μg/mL) (Gibco/Life Technologies), and l-glutamine (2 mM) (Life Technologies). Primary human bronchial epithelia cells (HBECs) were obtained from healthy donors and were differentiated at air–liquid interfaces (ALIs) as previously described^80^. For inhibitor studies, 2 × 10^5^ cells were seeded in 2 mL complete medium in six-well plates and incubated overnight at 37 °C/5% CO_2_. Cells were treated with phenelzine (400 µM; Sigma Aldrich) or GSK (400 µM) for 24, 48, and 72 h. For qRT-PCR analysis, at each time point, adherent cells were trypsinized, washed, and cell pellets stored at –80 °C until further processing. For microscopy studies, 4 × 10^4^ cells were seeded on coverslips and treated with inhibitors as described above. At each timepoint, coverslips were washed, fixed with 3.7% formaldehyde (Sigma Aldrich), and stored at 4 °C until processing. For SARS-CoV-2 studies, 2 × 10^5^ cells were seeded in six-well plates as described above and treated with inhibitors for 48 h prior to viral infection. After 1 h exposure, cells were replenished with fresh medium containing inhibitors and incubated at 37 °C/5% CO_2_ for a further 48 h. For qRT-PCR, cells were lysed in 1 mL Tri-reagent (Sigma Aldrich), while for microscopy studies, cells were fixed in 3.7% formaldehyde and collected by trypsinization and cell scraping.

### Patient specimen collection

Blood was collected from three COVID-19 patients admitted to the Royal Brisbane and Women’s Hospital (RBWH) and processed within 4 h of collection. PBMCs were isolated by density gradient centrifugation using SepMate^TM^ PBMC tubes (STEMCELL Technologies, Vancouver, Canada) according to the to manufacturer’s protocol. This study was performed in accordance with the NHMRC National Statement on Ethical Conduct in Human Research 2007 (updated 2018). Ethical approval was granted by the Royal Brisbane and Women’s Hospital Ethics Committee (HREC/2020/QRBW/63138) and accepted by the QIMR Berghofer (project ref# P3562).

### SARS-CoV-2 stock production and CCID_50_ titrations

SARS-CoV-2 infection studies at QIMR Berghofer were conducted in a dedicated PC3 (BSL3) suite, with safety approval from the QIMR Safety Committee (P3600). The SARS-CoV-2 isolate was kindly provided by the Queensland Health Forensic & Scientific Services, Queensland Department of Health, Brisbane, Australia. The virus (hCoV-19/Australia/QLD02/2020) was isolated from a patient and its sequence is deposited in GISAID (https://www.gisaid.org/; after registration and login, sequence can be downloaded from https://www.epicov.org/epi3/frontend#1707af). Virus stock was generated by infection of Vero E6 cells at multiplicity of infection (MOI) ≈ 0.01, with supernatant collected after 3 days, cell debris removed by centrifugation at 3000×*g* for 15 min at 4 °C, and virus aliquoted and stored at –80 °C. Virus titers were determined using standard CCID_50_ assays. The virus was determined to be *Mycoplasma* free using co-culture with a non-permissive cell line (i.e., HeLa) and Hoechst staining as described^81^. For CCID_50_ virus titration, Vero E6 cells were plated into 96-well flat-bottomed plates at 2 × 10^4^ cells per well in 100 µL of medium. Ten-fold serial dilutions of virus stock or samples were performed in 100 µL RPMI 1640 supplemented with 2% FCS. Hundred microliter of serially diluted samples were added to Vero E6 cells and the plates cultured for 5 days at 37 °C and 5% CO_2_. The virus titer was determined by the method of Spearman and Kärber (a convenient Excel CCID_50_ calculator is available at https://www.klinikum.uni-heidelberg.de/zentrum-fuer-infektiologie/molecular-virology/welcome/downloads).

### Western blot

For ACE2 and LSD1 membrane protein expression, cell surface protein extracts were prepared from Caco-2 cells (1 × 10^7^) using the PierceTM Cell Surface Protein Biotinylation and Isolation Kit (Life Technologies, Carlsbad, CA) according to manufacturer’s instructions and as previously described^82^. Cell membrane extracts were separated in a 4%–20% Mini-PROTEAN® TGX™ Precast Gel4 (Bio-Rad Laboratories, Hercules, CA) and transferred to nitrocellulose membranes. After blocking in 5% milk, membranes were incubated with primary antibodies: mouse monoclonal anti-ACE2 (Santa Cruz, sc-390851) or rabbit polyclonal anti-KDM1/LSD1 (Abcam, ab17721) overnight at 4 °C. Mouse monoclonal anti-integrin beta I (Abcam, ab30394) was used as a positive control for cell membrane protein extraction. Primary antibodies were detected using mouse and rabbit HRP antibodies and enhanced chemiluminescence reagents (Western Lightning ECL-Plus; Perkin-Elmer, Waltham, MA). Signals were imaged with the iBrightTM CL1500 Imaging System (Thermo Fisher Scientific, Waltham, MA).

### Immunoprecipitation

For LSD1 interaction studies with CoREST and HDAC2, total protein lysates were prepared from Caco-2 cells (1 × 10^7^) using RIPA buffer (20 mM Tris-HCl pH 7.5, 150 mM NaCl, 1 mM EDTA, 0.1% SDS, 1% NP40, 1× protease inhibitor cocktail). Cells were lysed for 30 min at 4 °C prior to LSD1 pulldown with 15 µg of rabbit polyclonal anti-KDM1/LSD1 (ab17721, Abcam) and Protein A magnetic beads (16-661, Merck Millipore). The beads were washed, and the bound proteins were eluted with immunoblot loading buffer containing beta-mercaptoethanol, followed by incubating at 95 °C for 5 min. Samples were subjected to western blotting as described above. Membranes were probed with rabbit anti-CoREST antibody (ab183711, Abcam) or rabbit anti-HDAC antibody (57156, Cell Signaling Technology) overnight at 4 °C, and detected using mouse and rabbit HRP antibodies and enhanced chemiluminescence reagents. Signals were imaged with the iBrightTM CL1500 Imaging System.

For ACE2 methylation studies, cell surface protein extracts were prepared from Caco-2 cells (1 × 10^7^) using the PierceTM Cell Surface Protein Biotinylation and Isolation Kit as described above. Membrane extracts were subjected to ACE2 pulldown with 15 µg rabbit anti-ACE2 antibody (SN0754) (NBP2-67692, Novus Biologicals) as described above, and western blot performed using the rabbit anti-pan methyl Lysine antibody (ab7315, Abcam).

### Dot blot assay

Dob blot analysis was performed on custom made ACE2me2 peptide (Mimotopes). Methylation was detected using rabbit anti-pan methyl Lysine antibody (ab7315, Abcam) and rabbit HRP-conjugated secondary antibodies. Signals were detected with enhanced chemiluminescence reagents (Western Lightning ECL-Plus; Perkin-Elmer, NEL104001) and the iBrightTM CL1500 Imaging System (Thermo Fisher Scientific).

### Plasmid transfection assay

Transfections were performed using the NEON^TM^ Transfection System kit (MPK5000; Invitrogen) to transfect Caco-2 cells with 15 μg of plasmid according to the manufacturer’s instructions. Vector only plasmid (pTracer-CMV/BSD, Thermo Fisher Scientific), LSD1 WT, ACE2 WT, ACE2 A31 (lysine 31 to alanine mutant), or ACE2 F31 (lysine 31 to phenylalanine mutant, hypermethylation mimic) were used for transfection. Transfected cells were incubated for 48 h followed by adding SARS-CoV-2 spike protein. After 1 h incubation, samples were subjected to the Duolink^®^ proximity ligation assay.

### LSD1 demethylation assay

LSD1 demethylation activity on custom made ACE2me2 peptides (Mimotopes) was assessed using the LSD1 fluorimetric drug discovery kit according to the manufacturer’s instructions (BML-AK544, Enzo Life Sciences). Briefly, ACE2me2 peptides were incubated with human recombinant LSD1 enzyme in LSD1/HRP assay buffer containing 1× HRP and 1× CELLestial^®^ Red Peroxidase substrate. For LSD1 inhibition, various LSD1 inhibitors were incubated with recombinant LSD1 enzyme for 30 min at room temperature prior to adding ACE2me2 peptide. The fluorescent signal was measured in kinetic mode (40 s intervals) at an excitation of 530–570 nm, emission of 590 nm for 5–30 min using the Synergy H4 multi-mode plate reader system (BioTek).

### Peptide design and synthesis

#### ACE201/ACE202 cell surface peptides

Glutamine 493 in the spike protein RBD binds to ACE2 and interacts with and binds to ACE2 at lysine 31. Despite the development of SARS-CoV-2 variants, the spike protein still targets ACE2 at lysine 31^83^, providing an ongoing rationale to target this interaction with a competitive peptide. Structural analysis in silico to predict^34^ methylation of lysine 31 showed that its methylation reduces the efficiency of interaction with the spike RBD at glutamine 493, with de-methylation increasing the interaction.

Using sequence analysis of this motif with SATPdb^84^ to identify optimal competitive peptide inhibitor matches, we designed several overlapping peptide sequences that mimicked this region and targeted the RBD interaction motif around lysine 31, and the overlapping de-methylation motif identified in silico^34^. These peptides were capped with acetyl and NH2 ends to more closely mimic the native protein charge and resist enzymatic degradation. Of the peptides with overlapping sequences selected to target this region, two lead candidates (ACE2-01, ACE2-02) were taken forward, both of which included lysine 31. These peptides competitively blocked the interaction between spike protein and lysine 31, either by interfering with the ACE2- spike protein interaction or by binding to the spike protein to produce an open-conformation decoy. These peptides also competitively blocked enzymatic access to lysine 31 as a decoy interaction, interfering with its de-methylation, which was achieved by mimicking the ACE2-spike interaction motify/lysine de-methylation motif, in which the LSD1 catalytic pocket or the RBD spike domain interacts with the peptide and not the target protein (ACE2-01 and ACE2-02).

#### NACE2i

We hypothesized that molecules mimicking the NLS of ACE2 would act as specific competitive inhibitors of ACE2 translocation from the cytoplasm to the nucleus. We identified a high affinity NLS domain (RKKKNK) within the ACE2 C-terminal tail using the NLS prediction tool NLStradamus^59^, which was embedded within a high scoring methylation/de-methylation motif identified using the Wen et al.^34^ tool. This sequence displayed high binding affinity for importin subunits, so we created an optimized myristoylated inhibitor based on a peptide sequence mimicking the native protein sequence. Both peptide length optimization and alanine walking to identify critical residues (data not shown) were carried out. The optimized myristoylated inhibitor (NACE2i) was predicted to be a more potent inhibitor of nuclear transport, and was designed to have inherent cell penetrating properties.

Peptide inhibitors were synthesized using automated modern solid-phase peptide synthesis and purification technology using the mild Fmoc chemistry method, for example as described in the ref. ^85^. Peptides were purified using automated preparative reversed-phase high-performance liquid chromatography (RP-HPLC). Fractions were analyzed using analytical RP-HPLC and mass spectrometry. Fractions of 98% purity or higher were combined to give the final product. Where required peptide constructs were also tagged with FAM5.

### Infection with SARS-CoV-2 in the presence of inhibitors

Caco-2 cells were tested negative for *Mycoplasma* contamination prior to experiments. Caco-2 cells were seeded at 2 × 10^5^ cells per well in six-well plates in DEME (10% FBS) and incubated overnight at 37 °C, 5% CO_2_. Forty-eight hours prior to infection, cells were treated with 400 μM phenelzine or 400 μM GSK. At the time of infection, plates were transferred to the BSL3 facility and inhibitor-containing medium was removed and replaced with SARS-CoV-2 virus inoculum (MOI 1) containing DEME (5% FBS). After 1 h incubation at 37 °C, 5% CO_2_, the virus inoculum was removed and cells were washed three times with PBS to remove unbound virus prior to adding inhibitor-containing medium. 48 h post infection (48 hpi), the cell culture supernatant and cells were collected separately in TRIzol reagent and RNA was extracted using the Direct-zol RNA miniprep kit (Zymo Research, Irvine, CA) following the manufacturer’s instructions. For ASI digital pathology and FACS, cells were fixed with 4% formaldehyde for 30 min at room temperature.

### Determination of viral titers by qRT-PCR

Viral titers (TCID_50_ equivalents per mL) in the extracted RNA were determined by qRT-PCR using a real-time fluorescent RT-PCR kit for detecting 2019-nCoV (BGI Genomics, China) following the manufacturer’s instructions. Positive control (mix of pseudo-virus with target virus genes and internal reference) and blank control (DNase/RNase free water) were used as quality control. The limit of detection was 100 copies/mL. The quantity of viral genomes was calculated by normalizing to a viral stock with a known viral titer.

### Flow cytometry

Fixed cells were washed with PBS and resuspended in PBS containing 1% BSA prior to antibody staining. Primary antibodies targeting LSD1 (ab17721, Abcam, Cambridge, UK), Coronavirus (FIPV3-70) (sc65653, Santa Cruz Biotechnology, Dallas, TX), and ACE2 (AF594 conjugated, sc390851, Santa Cruz Biotechnology) and secondary chicken anti-mouse AF488 (A21200, Thermo Fisher Scientific, Waltham, MA) and donkey anti-rabbit AF647 (A31573, Thermo Fisher Scientific) antibodies were used. For PBMC samples, CD3 (344838, BioLegend, San Diego, CA), CD4 (558116, BD Biosciences, San Jose, CA), CD8α (555366, BD Biosciences), CD45RA (304133, BioLegend), and CCR7 (353244, BioLegend) antibodies were used. Appropriate antibody controls were used for all flow cytometry stain and acquisition. All samples were acquired on an LSR Fortessa cytometer (BD Biosciences, Franklin Lakes, NJ), and data were analyzed using FlowJo v10 software.

### Immunofluorescent staining and analysis

IFA imaging and analysis was carried out using previously established and optimized protocols^50,86–88^. Cells were fixed with formaldehyde (3.7%) and then immuno-stained with antibodies targeting the viral spike and nucleocapsid proteins, LSD1, H3k4me2, H3k9me2, ACE2, and TMPRSS2. Cells were permeabilized by incubating with 0.5% Triton X-100 for 15 min, blocked with 1% BSA in PBS, and were probed with primary antibodies followed by visualization with secondary donkey anti-rabbit, mouse, or goat antibodies conjugated to Alexa Fluor 488, 568, or 647. Coverslips were mounted on glass microscope slides with ProLong Glass Antifade reagent (Life Technologies, Carlsbad, CA). For SARS-CoV-2 spike protein experiments, Caco-2 cells (8 × 10^4^) were seeded on coverslips for 48 h prior to treatment with 20 μL of purified SARS-CoV-2 spike protein S1 (Glu14-Ser680) containing a polyhistidine tag at the C-terminus (1.52 mg/mL) (kindly supplied by the University of Queensland Protein Expression Facility) for 24 h. Protein targets were localized by digital pathology laser scanning microscopy. Single 0.5 μm sections were obtained using an ASI Digital pathology (ASI Digital pathology is characterization of both the fluorescent intensity as per normal immunofluorescent imaging as well as the ability to count the population of cells positive or negative for antibodies, allow population dynamics to be investigation using powerful custom designed algorithms and automated stage. This also allows the imaging and counting of large cell numbers for statistical power) microscope using a 100× oil immersion lens running ASI software. The final image was obtained by averaging four sequential images of the same section. Digital images were analyzed using automated ASI software as described previously^63^ (Applied Spectral Imaging, Carlsbad, CA) to determine the distribution and intensities automatically with automatic thresholding and background correction of the average nuclear fluorescence intensity (NFI), allowing for the specific targeting of expression of proteins of interest. Digital images were also analyzed using ImageJ software (ImageJ, NIH, Bethesda, MD, USA) to determine the total cell fluorescence or cell surface only fluorescence for non-permeabilized cells. ImageJ software with automatic thresholding and manual selection of regions of interest (ROIs) was used to calculate the Pearson’s co-efficient correlation (PCC) for each pair of antibodies. PCC values range from: −1 = inverse of co-localization, 0 = no co-localization, +1 = perfect co-localization. The Mann–Whitney nonparametric test was used to determine significant differences between datasets.

Appropriate controls were used for all experiments including no antibody controls, primary only, or secondary only controls in both IFA and Duolink assays. Lamin B1 (nuclear/intracellular only) and integrin-beta I (cell surface/cytoplasm only) were employed to confirm the fidelity of our permeabilization protocols by confirming the signal seen for our antibodies in either permeabilized or non-permeabilized samples as detecting the specific target of interest. Non-permeabilized samples were used to detect cell surface proteins and permeabilized samples were used to detect intracellular proteins using previously established protocols. Primary CD8^+^ T cells with either negative or low expression of LSD1 were used as a negative control for cell surface expression of LSD1. For ACE2, MRC5, which low or no expression of ACE2, was used as a negative control for ACE2 antibodies.

ANDOR WD Revolution super-resolution imaging was carried out according to normal specifications for the system with sample preparation identical to that for immunofluorescence.

### Proximity ligation assay

The Duolink proximity ligation assay was employed using PLA probe anti-mouse PLUS (DUO92001), PLA probe anti-rabbit MINUS (DUO92005), and Duolink In Situ Detection Reagent Red Kit (DUO92008) (Sigma Aldrich). Cells were fixed, permeabilized, and incubated with primary antibodies targeting LSD1 and ACE2. Cells were processed according to the manufacturer’s recommendations. Finally, coverslips were mounted onto slides and examined as above.

### Bioinformatics analysis tools for predicting PTMS

For identifying lysine residues susceptible to methylation and demethylation, the bioinformatics analysis tools from Wen et al.^35^. were used, which employed a support vector machine algorithm cut-off of 0.5. Any score above 0.5 was considered highly likely to be a lysine positive for methylation/de-methylation.

### Fluorescence polarization

Fluorescence polarization assays were performed using the CLARIOstar Plus plate reader (BMG Labtech) with the fluorescein-Ahx-tagged ACE2 peptide sequence ^766^RDRKKKNKARSGEN^779^ manufactured by GeneScript Biotech (Piscataway, NJ) and recombinantly expressed importin protein. Each assay contained 200 nM ACE2 FITC and two-fold serially-diluted IMPα (starting concentration 100 µM) across ten wells to a total volume of 200 µL. Fluorescence polarization readings were taken using 96-well black Fluotrac microplates (Greiner Bio-One; Kremsmünster, Austria). Assays were repeated in triplicate and contained a negative control (no binding partner) and blank (Tris buffered saline, pH 8). Triplicate data was fitted to a single binding curve using GraphPad Prism.

### Microscale thermophoresis

A Monolith NT.115 instrument (NanoTemper Technologies) was used to perform microscale thermophoresis (MST). Binding assays involved mixing 200 nM of FITC-Ahx-tagged ACE2 peptide (Genscript, Piscataway, NJ; includes the NLS residues ^766^RDRKKKNKARSGEN^779^), with 2-fold serially diluted, purified importin-α ΔIBB (commencing at 70 µM) in 20 mM HEPES, 125 mM NaCl, pH 8.0. Samples were applied to Monolith NT Premium Capillaries (NanoTemper Technologies; München, Germany), and thermophoresis was assessed at 25 °C with laser off/on/off times of 5/30/5 s. Experiments were conducted at 40% LED power and 40% MST infrared laser power. Triplicate data was fitted to a single binding curve using GraphPad Prism.

### Protein expression and purification

The importin-α ΔIBB (lacking the autoinhibitory domain) gene, encoding residues 71–529, was cloned in the pET30 vector as described previously^89,90^. Recombinant BL21(DE3) pLysS was expressed in *Escherichia coli* cells transformed with the target plasmids (either vector only, wild-type ACE2 or mutant ACE2 with a hypermethylation mimic (ACE2 K31F) replacing lysine 31 (phenylalanine) (including a 6× His tag)) using the auto-induction method^91^. After expression for 24 h at room temperature, cells were harvested by centrifugation. The resulting pellet was resuspended using buffer A (20 mM imidazole, 300 mM NaCl, 50 mM phosphate, pH 8.0) and subjected to three freeze-thaw cycles and treatment with lysozyme and DNase. Purification was undertaken using a 5 mL HisTrap column (GE Healthcare, Chicago, IL) incorporating a 15-column volume wash step with buffer A followed by a 5-column volume gradient elution step with buffer B (500 mM imidazole, 300 mM NaCl, 50 mM phosphate, pH 8.0). The sample was subjected to further purification using Superdex 200 pg 26/600 gel filtration chromatography in Tris-buffered saline pH 8 (50 mM Tris, pH 8, 125 mM NaCl). The homogenous peak corresponding to importin-α was collected and concentrated 0.4 mM using a 10 kDa Amicon centrifugal filter device.

### Crystallization and data collection

FITC-Ahx-tagged ACE2 peptide was mixed with importin alpha protein (0.4 mM) in a 1:3 protein to peptide molar ratio. Hanging drop vapor diffusion was used for crystallization, and the peptide-protein mixture was mixed 1:1 v/v with reservoir solution. Yellow rod-shaped crystals were obtained using a reservoir solution of 0.65 M sodium citrate and 0.1 M sodium HEPES, pH 7.5, 0.01 M DTT. Crystals were cryo-protected with 20% glycerol and diffracted at the Australian Synchrotron MX2 beamline^92^. All data reduction and integration were performed using iMosflm^93^ and merging and scaling in Aimless^94^. Molecular replacement in PhaserMR^95^ using PDB 6BW0 as the search model^90^ was used to build a preliminary structure, and the final structure was generated using iterative cycles of manual building in Coot^96^ and refinement in Phenix^97,98^. The final structure was validated and deposited to the Protein Data Bank under the accession code 7JVO.

### Electrophoretic mobility shift assay

FITC-Ahx-tagged ACE2 peptide (300 µM) was mixed with importin-α ΔIBB protein (430 µM) and electrophoresed through a 0.75% agarose gel in TB Buffer (45 mM boric acid, 45 mM Tris base, pH 8.0) for 1 h at 40 V. ACE2 peptide alone and importin-α ΔIBB alone were used controls. The gel was first imaged under UV light using a Gel Doc XR + system before being stained using Coomassie brilliant blue.

### RNA-seq

Sequence reads were trimmed for adapter sequences using Cutadapt 1.9^99^ and aligned using STAR 2.5.2a^100^ to a custom hybrid genome consisting of the human genome assembly GRCh37 Ensembl release 70 together with SARS-CoV-2 (GenBank NC_045512.2), where the SARS-CoV-2 genome was represented as a separate “gene”. Quality control metrics were computed using RNA-SeQC 1.1.8^101^ and transcript abundances were quantified using RSEM 1.2.30^102^.

Downstream analysis of the RNA-seq data mapped to the custom hybrid gene model was performed using R version 3.5.1. The SARS-CoV-2 “gene” and protein-coding genes with greater than three counts per million in three or more samples were kept for further analysis. Trimmed mean of *M*-values (TMM) normalization and differential gene expression analysis were performed using edgeR^103^. The prcomp function in R was used to perform principal component analysis on gene-wise centered and scaled values of TMM-normalized expression data, and the removeBatchEffect function from limma^104^ was applied prior to PCA to remove the batch effect from the expression data for visualization purposes only. For differential expression analysis, an additive linear model was used to incorporate batch and treatment. Differentially expressed genes (DEGs) between the control group and each of the treated groups were determined using absolute log2 fold change (logFC) > 1 and a false discovery rate (FDR) < 0.01. To perform pathway analysis, the bitr function from clusterProfiler^105^ was used to convert gene IDs from Ensembl to Entrez and then subsequently passed to the enrichPathway function in ReactomePA^106^ before plotting the results with the dotplot function in clusterProfiler^105^. Heatmaps were produced using Heatmap from the ComplexHeatmap package^107^. Euler diagrams were generated using the euler function from the eulerr package^108^. Scatterplots and bar charts were constructed using ggplot2^109^. To estimate virus replication levels in infected cells, sequence reads were aligned to SARS-CoV-2 only, and samtools 1.9^110^ was used to estimate the mapping rate of the reads to the viral genes.

### Statistical analysis

Statistical analyses were performed with GraphPad Prism 8 software (GraphPad Software, San Diego, CA). One-way analysis of variance (ANOVA) with Dunnett’s post-test was used to test statistical significance. Only *P*-values < 0.05 were considered statistically significant. Where applicable, statistical significance is denoted by ^∗^*P* < 0.05, ^∗∗^*P* < 0.01, ^∗∗∗^*P* < 0.001, and ^∗∗∗∗^*P* < 0.0001. Data are expressed as mean ± SEM.

## Supplementary information

Supplementary Information

## Data Availability

Protein Data Bank files associated with the structures generated in this study have been deposited to the Protein Data Bank and issued PDB Accession code 7JVO. All datasets are available from the authors upon request.
