# Author Correction: Targeting novel LSD1-dependent ACE2 demethylation domains inhibits SARS-CoV-2 replication

**DOI:** 10.1038/s41421-021-00308-8

**Published:** 2021-07-27

**Authors:** Wen Juan Tu, Robert D. McCuaig, Michelle Melino, Daniel J. Rawle, Thuy T. Le, Kexin Yan, Andreas Suhrbier, Rebecca L. Johnston, Lambros T. Koufariotis, Nicola Waddell, Emily M. Cross, Sofiya Tsimbalyuk, Amanda Bain, Elizabeth Ahern, Natasha Collinson, Simon Phipps, Jade K. Forwood, Nabila Seddiki, Sudha Rao

**Affiliations:** 1 Gene Regulation and Translational Medicine Laboratory, QIMR Berghofer Medical Research Institute, Brisbane, QLD Australia; 2 The Inflammation Biology Group, QIMR Berghofer Medical Research Institute, Brisbane, QLD Australia; 3 Medical Genomics, QIMR Berghofer Medical Research Institute, Brisbane, QLD Australia; 4 School of Biomedical Sciences, Charles Sturt University, Wagga Wagga, NSW Australia; 5 Department of Medical Oncology, Monash Health, Clayton, VIC Australia; 6 School of Clinical Sciences, Monash University, Clayton, VIC Australia; 7 Molecular Parasitology Laboratory, QIMR Berghofer Medical Research Institute, Brisbane, QLD Australia; 8 Respiratory Immunology Laboratory, QIMR Berghofer Medical Research Institute, Brisbane, QLD Australia; 9 U955, Equipe 16, Créteil, France; 10 Université Paris-Est Créteil, Faculté de médecine, Créteil, France; 11 Vaccine Research Institute (VRI), Créteil, France; 12 INSERM U1184, CEA, IDMIT Department, Immunology of Viral, Auto-Immune, Hematological and Bacterial Diseases (IMVA-HB), Université Paris-Saclay, 92265 Fontenay-aux-Roses, France

Correction to: *Cell Discovery* (2021) **7**:37

10.1038/s41421-021-00279-w, published online 24 May 2021

In the original publication of this Correspondence^1^, there is a missing affiliation for Dr. Nabila Seddiki. The corrected affiliations for Dr. Nabila Seddiki should be as follows:

^9^U955, Equipe 16, Créteil, France. ^10^Université Paris-Est Créteil, Faculté de médecine, Créteil, France. ^11^Vaccine Research Institute (VRI), Créteil, France, ^12^INSERM U1184, CEA, IDMIT Department, Immunology of Viral, Auto-Immune, Hematological and Bacterial Diseases (IMVA-HB), Université Paris-Saclay, 92265 Fontenay-aux-Roses, France.

This correction does not affect the description of the results or the conclusion of this work.

In the original publication of this Correspondence^1^, there was a mistake in Fig. 2o. There was a mathematical error in the subtraction of background for the TCID_50_ assay. We have now corrected the error in background subtraction and have replotted the graph. We have engaged an independent statistician to validate the updated graph. This correction does not alter the overall results or the conclusions of this work.

**Fig. 2:**
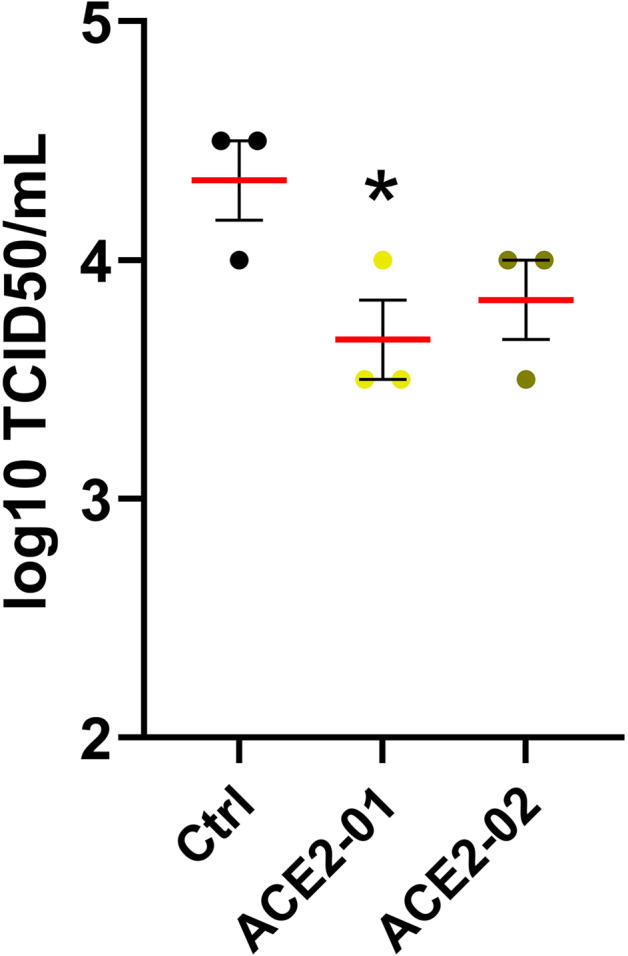
**o** TCID50 assay to measure infectious viral titers in the culture. Data represent mean ± SEM, *n* = 3. Unpaired *t*-test, *P < 0.05 denotes significant differences.
