# Supplementary material for: Targeting novel LSD1-dependent ACE2 demethylation domains inhibits SARS-CoV-2 replication
Source: Cell Discov. 2021 May 24;7:37. doi: 10.1038/s41421-021-00279-w (PMC8143069; doi:10.1038/s41421-021-00279-w)
Supplement: Supplementary file 1 — Supplementary Information [file 41421_2021_279_MOESM1_ESM.pdf]

SUPPLEMENTARY INFORMATION

a

LSD1 Residues	Orientation	Score
65-82	O-I	1392
313-336	I-O	716
502-522	O-I	772
809-826	I-O	3438

G9A Residues	Orientation	Score
896-912	I-O	669

Cut-off score of >500 indicates strong transmembrane motif

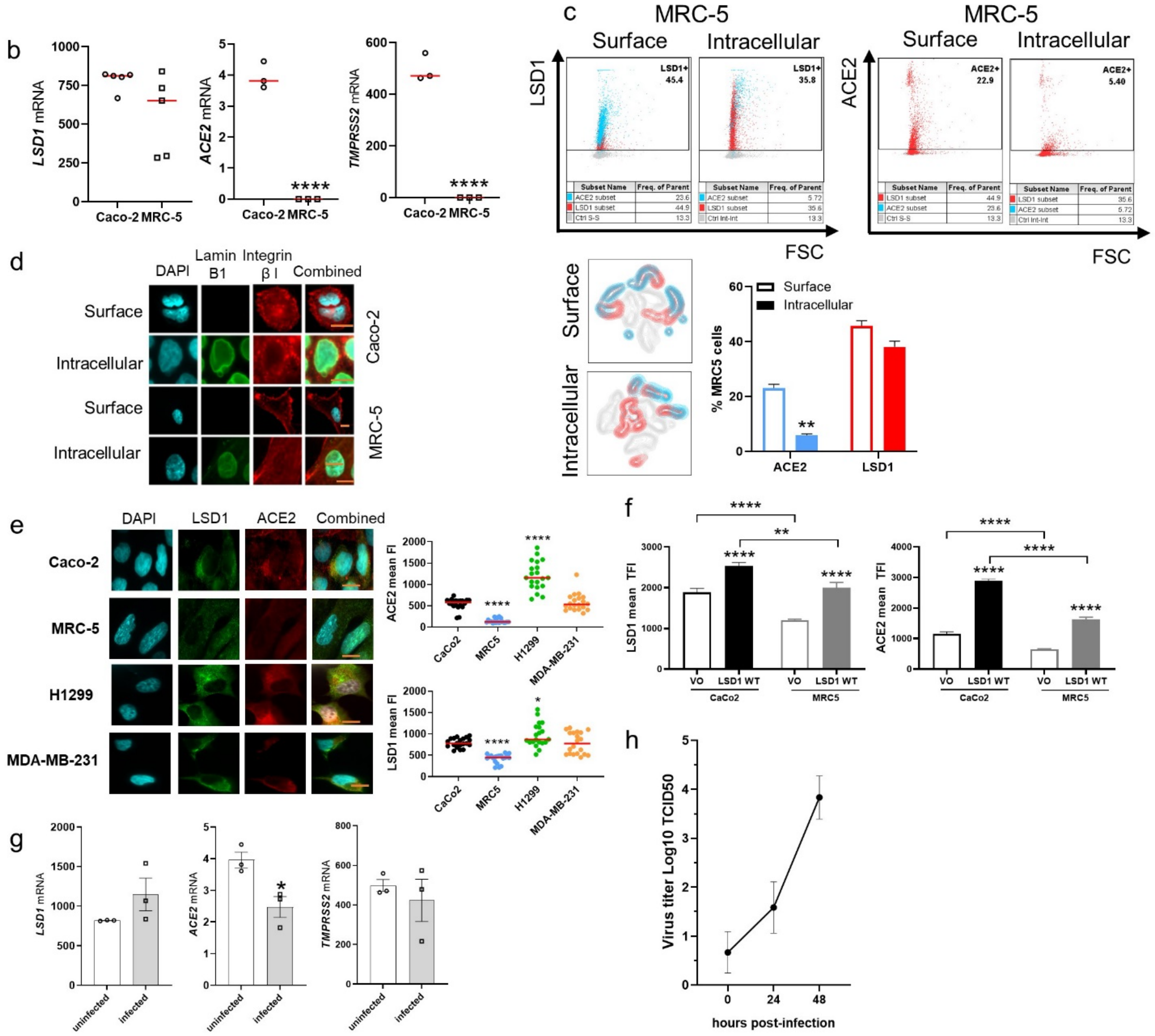

Supplementary Figure S1

a The TMpred transmembrane prediction tool[1] was used to examine LSD1, G9A, and SETDB1 for transmembrane regions. Only scores ≥500 are considered significant. Orientation is indicated as O-I (out to in) or I-O (in to out) in regards to outside or inside cell membrane. Specific transmembrane regions with associated residue scores are indicated in table form.

b qRT-PCR analysis of *LSD1*, *ACE2,* and *TMPRSS2* mRNA expression in Caco-2 and MRC-5 cells. Data are mean ± SEM. (n=3, unpaired t test: **** P <0.0001).

c FACS tSNE analysis of cell surface and intracellular expression of ACE2 and LSD1 in MRC-5 cells. The bar graph indicates the percentage $ACE2^+$ or $LSD1^+$ cells in the total MRC-5 population, also shown in the FACS dot plots ($ACE2^+$ cells in blue and $LSD1^+$ cells in red). Data are mean ± SEM (n=3).

d Representative image of uninfected Caco-2 and MRC-5 cells using the ASI system. Cells were either not permeabilized (surface) or permeabilized (intracellular) for immunostaining for lamin β1 and integrin β1. DAPI (blue) was used to visualize nuclei. Scale bar, 12 μm (inset).

e Representative image of uninfected Caco-2, MRC-5, H1299, and MDA-MB-231 cells using the ASI system. Cells were not permeabilized (surface) for immunostaining for LSD1 and ACE2. DAPI (blue) was used to visualize nuclei. Scale bar, 12 μm (inset). Dot plot quantification of the fluorescence intensity (cell surface) of ACE2 and LSD1 in Caco-2, MRC-5, H1299, and MDA-MB-231 cell lines. >50 cells were analyzed for each group. Mann-Whitney test: ∗ $P < 0.05$, ∗∗∗∗ $P < 0.0001$.

f Dot plot quantification of the fluorescence total intracellular intensity of ACE2 and LSD1 in Caco-2 and MRC-5 cells transfected with vector only (VO) and LSD1 overexpressing wild-type (WT) plasmids for 24 h. >50 cells were analyzed for each group. Data are mean ± SEM. Mann-Whitney test: ∗∗ $P < 0.01$, ∗∗∗∗ $P < 0.0001$.

g qRT-PCR analysis of *LSD1*, *ACE2,* and *TMPRSS2* mRNA expression in uninfected (depicted in Figure 1) versus SARS-CoV-2-infected Caco-2 cells. (n=3, unpaired t test: * p<0.05).

h $TCID_{50}$ assay to measure infectious viral titers in the culture supernatants of SARS-CoV-2-infected human biliary epithelial cells (HBECs). Data represent mean ± SEM, n = 3. One-way ANOVA, ∗∗ $P < 0.01$, denote significant differences.

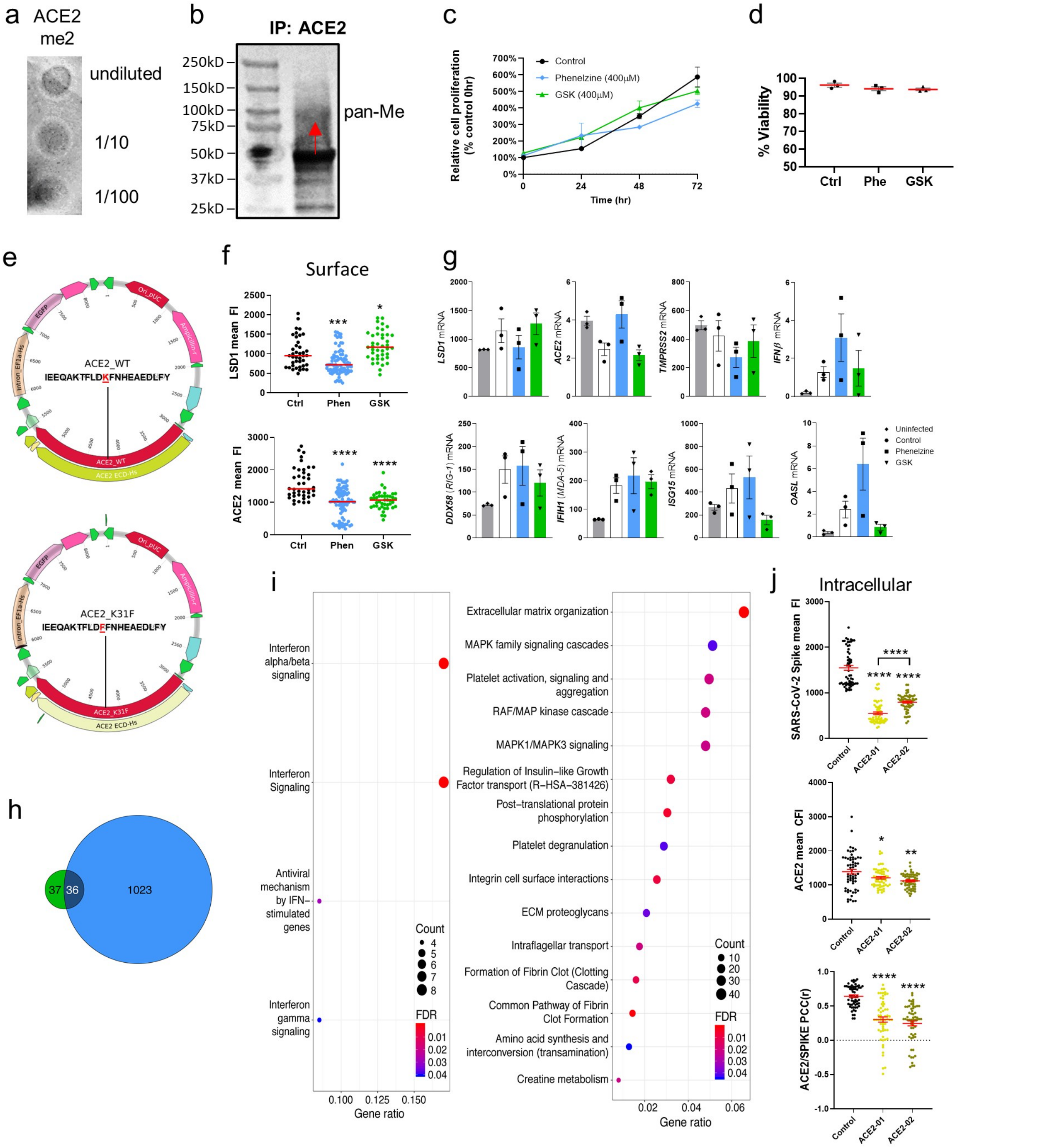

Supplementary Figure S2

a ACE2-Me2 peptide at different concentrations (undiluted, 1/10 dilution, or 1/100 dilution) were treated with a recombinant LSD1 enzyme and subjected to dot blot analysis with pan-methylation lysine antibody. Images depict example dot blots for n=3 dot blots.

b Western blot analysis of ACE2 IP samples. Following ACE2 IP of Caco-2 cell membrane extract lysates, samples were analyzed by SDS-PAGE and blotted for pan-methylation lysine.

c Cell proliferation analysis of Caco-2 cells following treatment with/without phenelzine or GSK over a 72 h period. Proliferation was analyzed using WST-1 reagent and absorbance read after 2 h incubation. The graph depicts relative cell proliferation from three replicates expressed as a percentage of control cells (untreated, 0 h). Statistical significance was calculated using one-way ANOVA at each time point.

d FACS analysis of cell viability in uninfected Caco-2 cells with phenelzine and GSK treatments (n=3).

e Depicted are the ACE2 WT, ACE2 A31 (lysine 31 to alanine mutant), and ACE2 F31 (lysine 31 to phenylalanine mutant (hypermethylation mimic)) plasmids.

f Dot plot quantification of the fluorescence intensity (cell surface) of ACE2 and LSD1 in SARS-CoV-2-infected Caco-2 cells with phenelzine or GSK treatment. >50 cells were analyzed for each group and were quantified with a digital pathology assay (ASI system). Mann-Whitney-test: ** $P < 0.01$, **** $P < 0.0001$.

g qRT-PCR analysis of *LSD1*, *ACE2*, *TMPRSS2, IFNα*, *IFNβ*, *DDX58 (RIG-1)*, *IFIH1 (MDA-5)*, *ISG15,* and *OASL* mRNA expression in uninfected versus SARS-CoV-2-infected control (depicted in Figure 2), phenelzine (400 μM), and GSK (400 μM) treated Caco-2 cells 48 hpi. The graph illustrates the number of gene transcripts from three replicates normalized to the geometric mean of *HPRT1*, *GAPDH,* and *ACTB*. Statistical significance was calculated using one-way ANOVA. NS, not significant.

h Euler diagram comparing DEGs from GSK vs. control (green) and phenelzine vs. control (blue).

i Dot plot visualization of enriched Reactome pathways for GSK vs. control (left) and phenelzine vs. control (right). The dot color represents the false discovery rate (FDR) value for each enriched Reactome pathway and size represents the gene ratio.

j Dot plot quantification of the fluorescence intensity (cytoplasmic) of SARS-CoV-2 spike and protein ACE2 in SARS-CoV-2-infected CaCo-2 cells with ACE2-01 or ACE2-02 treatment, >50 cells were analyzed for each group and were quantified by a digital pathology assay (ASI system). The PCC was calculated to assess colocalization (n = 20 cells were analyzed). Mann-Whitney test: ∗ $P < 0.05$, ∗∗ $P < 0.01$, ∗∗∗∗ $P < 0.0001$ denote significant differences.

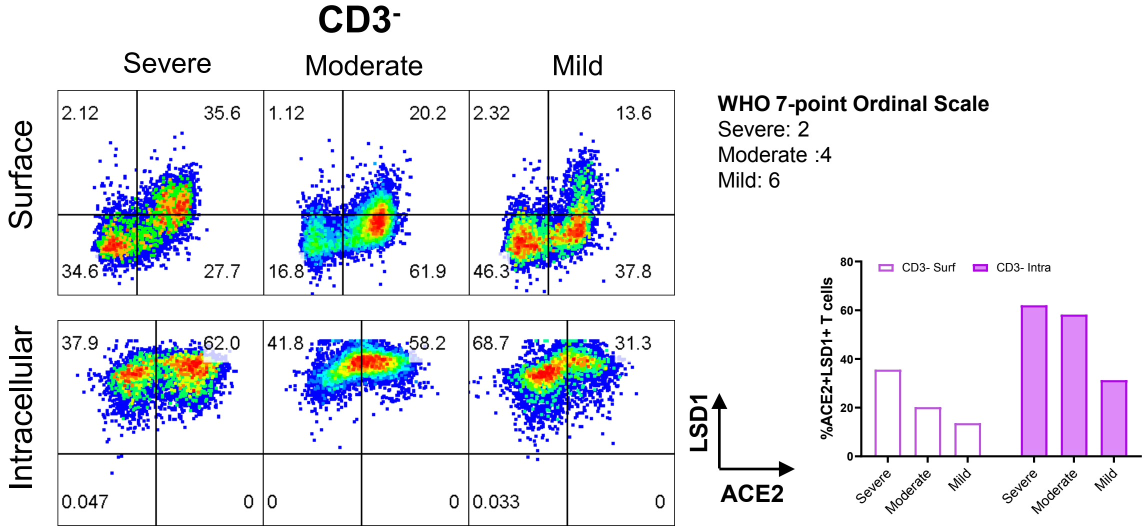

Supplementary Figure S3

FACS analysis of cell surface and intracellular expression of ACE2 and LSD1 in $CD3^-$ cells from COVID-19 patients (n=3). The bar graph indicates the percentage $ACE2^+LSD1^+$ cells in the total $CD3^-$ population.

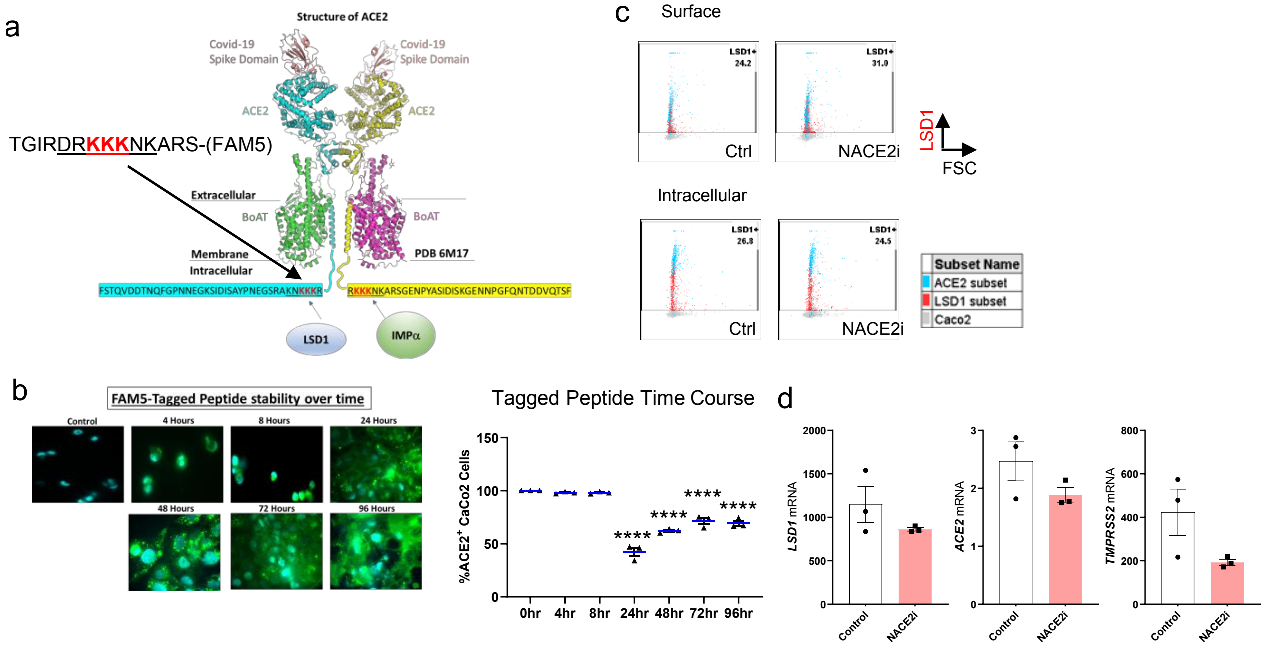

Supplementary Figure S4

a Development of a competitive peptide inhibitor targeting the C-terminal NLS region of ACE2 that also interacts with LSD1 and IMPα. This competitive peptide inhibitor also targets the region (in red) that is targeted for demethylation by LSD1. The peptide was tagged with a fluorescent tag (FAM5).

b Representative images of Caco-2 cells imaged with the ASI digital pathology system are shown, treated with the novel NACE2i (with FAM5 tag) to demonstrated stability of the novel ACE2 inhibitor over time in culture and target cells.

c FACS tSNE analysis of cell surface and intracellular expression of ACE2 and LSD1 in Caco-2 cells treated with NACE2i for 48 h. $ACE2^{+}$ cells in blue and $LSD1^{+}$ cells in red. Data are mean ± SEM (n=3).

d qRT-PCR analysis of *LSD1*, *ACE2,* and *TMPRSS2* mRNA expression in SARS-CoV-2 infected control (depicted in Figure 2) versus NACE2i-treated Caco-2 cells 48 h post infection. The graph illustrates the number of gene transcripts from three replicates normalized to the

geometric mean of *HPRT1*, *GAPDH,* and *ACTB*. Statistical significance was calculated using an unpaired t-test. NS, not significant.

Supplementary Table S1 Summary of ACE2me2 peptide sequence

ACE2 Peptide	Sequence	Length
ACE2me2	QAKTFLD{Lys(Me2)}FNHEAED	15
PEP1	TGIRDR{Lys(Me2)}KKNKARS	14
PEP2	TGIRDRK{Lys(Me2)}KNKARS	14
PEP3	TGIRDRKK{Lys(Me2)}NKARS	14
PEP4	TGIRDR{Lys(Me2)}K{Lys(Me2)}NKARS	14

Supplementary Table S2 Summary of IMPα2:ACE2 interactions. Total buried interface: 657.5 Å^2

Hydrogen Bonds			
#	ACE2	Dist. (Å)	IMPα2
1	B:LYS 770[NZ]	2.98	A:THR 155[OG1]
2	B:LYS 770[NZ]	3.10	A:GLY 150[O]
3	B:LYS 770[NZ]	2.87	A:ASP 192[OD1]
4	B:LYS 771[N]	2.82	A:ASN 188[OD1]
5	B:LYS 771[NZ]	2.88	A:ASN 228[OD1]
6	B:ASN 772[ND2]	3.18	A:SER 149[OG]
7	B:LYS 773[N]	2.91	A:ASN 146[OD1]
8	B:LYS 773[NZ]	3.03	A:GLN 181[OE1]
9	B:ARG 768[O]	3.53	A:TRP 231[NE1]
10	B:LYS 769[O]	2.91	A:ASN 235[ND2]
11	B:LYS 771[O]	2.87	A:TRP 184[NE1]
12	B:LYS 771[O]	3.09	A:ASN 188[ND2]
13	B:LYS 773[O]	2.97	A:TRP 142[NE1]
14	B:LYS 773[O]	3.00	A:ASN 146[ND2]
Salt Bridges			
1	B:LYS 770[NZ]	2.87	A:ASP 192[OD1]

Reference

1 Hofmann K. TMbase-A database of membrane spanning proteins segments. *Biol Chem Hoppe-Seyler* 1993; **374**:166.
